# Designing amendments to improve plant performance for mine tailings revegetation

**DOI:** 10.1002/agg2.20409

**Published:** 2023-09-01

**Authors:** Mark G. Johnson, David M. Olszyk, Tamotsu Shiroyama, Michael A. Bollman, Maliha S. Nash, Viola A. Manning, Kristin M. Trippe, Donald W. Watts, Jeffrey M. Novak

**Affiliations:** 1 U.S. Environmental Protection Agency, Pacific Ecological Systems Division, Center for Public Health and Environmental Assessment, Corvallis, Oregon, USA; 2 National Asian Pacific Center on Aging, Senior Environmental Employment Program, Corvallis, Oregon, USA; 3 U.S. Environmental Protection Agency, Pacific Ecological Systems Division, Center for Public Health and Environmental Assessment, Newport, Oregon, USA; 4 USDA ARS, National Forage Seed Production Research Center, Corvallis, Oregon, USA; 5 USDA ARS, Coastal Plain Soil, Water and Plant Conservation Research, Florence, South Carolina, USA

## Abstract

To provide recommendations for establishment of plants on low-pH Formosa Mine tailings, two greenhouse experiments were conducted to evaluate the use of remedial amendments to improve the survival and growth of Douglas fir (*Pseudotsuga menziesii*) seedlings. A preliminary experiment indicated that 1% lime (by weight) raised tailings pH, permitting seedling survival. However, high rates of biosolid application (BS; 2% by weight) added to supply nutrients were phytotoxic when added with lime. A gasified conifer biochar (BC) added to tailings at 1%, 2.5%, or 5% (by weight), along with lime and BS, caused an additional increase in pH, decreased electrical conductivity (EC), and tended to increase the survival of Douglas fir. The addition of a locally sourced microbial inoculum (LSM) did not affect survival. A subsequent experiment expanded our experimental design by testing multiple levels of amendments that included lime (0.5% and 1% by weight), three application rates (0.2%, 0.5%, and 2%) of two nutrient sources (BS or mineral fertilizer), BC (0% and 2.5%), and with or without LSM. There were many interactions among amendments. In general, Douglas fir survival was enhanced when lime and BC were added. These experiments suggest that amending with lime, a nutrient source, and BC would enhance revegetation on low-pH, metal-contaminated mine tailings.

## 1 ∣ INTRODUCTION

In the United States, there are an estimated > 160,000 abandoned mine lands, of which 565 are listed as superfund sites (https://www.epa.gov/superfund/abandoned-mine-lands; https://semspub.epa.gov/work/HQ/176021.pdf). In the Western United States, over 33,000 abandoned sites have a degraded environment with contaminated ground and surface water and heavy-metal-contaminated mine tailings (Mittal, 2011). Revegetation is required at these sites to prevent erosion of the heavy-metal-contaminated mining residuals into nearby waters.

Successful growth of plants in mine tailings is often limited by unfavorable mineral composition and low pH, high concentrations of soluble salts, lack of essential plant nutrients such as N and P, low microbial diversity, toxic concentrations of metals such as Cu and Zn, and, in some cases, the presence of polycyclic aromatic hydrocarbons and polychlorinated biphenyls (Gajic et al., 2018). Thus, remediation of these residuals is needed to promote the establishment and survival of beneficial plant cover. A successful remediation plan would consider multiple amendments to address site limitations that inhibit plant growth.

Acidic soils routinely receive supplements to raise the pH to a level adequate for plant growth. Liming materials usually consist of Ca and or Mg carbonates, oxides, and hydroxides that neutralize H+ in solution and remove the H+ from exchange sites (USDA, 1999). Liming materials have often been used to increase the pH of acid mine water (Potgieter-Vermaak et al., 2006) and to mitigate acid generation in tailings (Davis et al., 1999).

Biosolids (BS) have been used to improve degraded mine soils by providing essential elements for plants, supplying organic matter for soil microbes, and improving soil structure and moisture-holding capacity (EPA, 2000; Wijesekara et al., 2016). Potential drawbacks of BS include the presence of heavy metals and salts (Robledo et al., 2010), high electrical conductivity (EC) (Jordán et al., 2016), and pathogens and contaminants (Wijesekara et al., 2016). As an alternative to BS, mineral fertilizers (F) can also be used to improve soil fertility for waste rock revegetation, but in at least one study they did not perform as well as organic F, especially to provide plant-available P (Meikle et al., 1999).

The addition of inorganic and organic amendments can improve soil conditions and support plant establishment; however, a sustainable, long-term solution requires a functioning soil microbial community to drive nutrient cycling. Although microbial communities can develop over time, this process is generally slow (Cutler et al., 2014; Grman et al., 2015; Uroz et al., 2014). To accelerate the formation of soil microbial communities, several studies have examined the use of microbial inoculum in the form of soil transplants, commercially available synthetic consortia, and locally sourced microbial inoculum (LSM). While the functional capacity can be preserved by applying inoculum (Kumaresan et al., 2017), such inoculum has had mixed success in establishing microbial communities and affecting plant growth for potential usefulness in ecosystem restoration, including mine soils and tailings (Frewert et al., 2022; Li et al., 2023; Middleton & Bever, 2012; Moreira-Grez et al., 2019; Trippe et al., 2021; Wong et al., 2022; Wubs et al., 2016).

While lime would be the primary agent to increase soil pH, and BS or F can serve as sources of plant nutrients, biochar (BC) has a limited potential to raise pH and supply nutrients (Chintala et al., 2014; Hou et al., 2022). The potential benefits of BC may be increased if combined with other amendments such as compost to improve soil properties (Agegnehu et al., 2017). BC, a carbon-rich byproduct of pyrolysis of organic feedstocks (Lehmann & Joseph, 2015), has been proposed as a key amendment for land reclamation on mine tailings and spoils (Fellet et al., 2011; Ghosh & Maiti, 2020,^2021^; Ippolito et al., 2019). BC enhances or replaces other soil amendments by impacting soil hydrology (Masiello et al., 2015) (though not necessarily for sandy soils [Jeffrey et al., 2015]), influencing microbial community composition (Thies et al., 2015), and reducing soil salinity (Huang et al., 2019). While it is possible to create an acidic BC to lower the pH of a soil (Ippolito et al., 2016), BC often has some liming potential that can immobilize and reduce the bioavailability of heavy metals by increasing pH (Beesley et al., 2015) in soil (Dai et al., 2018; Wang et al., 2021). However, the acid-neutralizing effects of BC may be limited and depend on its calcium carbonate equivalence. The acid-neutralizing capacity of BC also may become limited over time, for example, Jones et al. (2012) showed that after 3 years, the acid-neutralizing potential of BC in acidic agricultural soil was fully depleted. In soils with high residual acidity, like mine tailings, liming effects must be sustained for longer periods of time to ensure plant survival and to promote ecological restoration (Edenborn et al., 2015). In addition, BC application can have adverse effects on the soil environment in some situations including reduced available water, increased soil salinity, adverse pH-related impacts, reduced availability of plant nutrients, toxicity of substances in BC, and changes in soil biota (reviewed in Brtnicky et al., 2021). All these potential effects depend on the characteristics of the tailings and BC and may be mitigated by careful pairing of soil deficiencies and BC attributes (Brtnicky et al., 2021).

The Formosa Mine Superfund site in southwestern Oregon (42.851271 N, −123.38583 W) is 16 km south of Riddle, OR, USA (Novak et al., 2018) and was primarily a copper and zinc mine with trace gold and silver (EPA, 2016). After mining ended in 1993, mine residuals (e.g., tailings) were left on the soil surface. In 2016, the U.S. Environmental Protection Agency in their final record of decision concluded that “…phytoremediation was not technically feasible for site application because of high contaminant concentrations, large extent and volume of mine materials and soils, excessive depth of contamination, and the heterogeneous nature of the mine materials matrix.” However, the record of decision did not consider recent developments in the use of remedial amendments, especially BC, which may facilitate plant establishment and growth. In a preliminary study, Ippolito et al. (2019) indicated that the Formosa Mine tailings had extreme acidification and Mehlich-3-extractable Cu and Zn between 61 and 68 mg·kg^−1^. Thus, a reclamation plan was recommended to first neutralize the acidic conditions and decrease plant-available Cu and Zn, followed by adding BC and a mixture of compost and/or manure to increase pH, soil organic carbon, and microbial growth (Ippolito et al., 2019).

Greenhouse studies have been performed to determine the impact of amendments on Formosa Mine tailings on grass species growth and metal immobilization. Both lime and BC alone decreased leachable metals (Ippolito et al., 2019; Phillips et al., 2016), and while lime is expedient for alleviating low-pH issues important for plant establishment, it has also been shown that plants can establish in tailings amended with high rates of BC alone (Phillips et al., 2016). When BC and lime were combined, there was an increase in pH over lime alone (Ippolito et al., 2019; Novak et al., 2018), but studies differed on the impact on aboveground biomass (Frewert et al., 2022; Ippolito et al., 2019). Shoot and root biomass increased for blue wildrye (*Elymus glaucus* Buckley ssp. *jepsonii* [Burtt Davy] Gould) only with lime with no effect of BC (Novak et al., 2018). The accumulation of some heavy metals in root and shoot tissue decreased for rye (*Secale cereale* L.) and *E. glaucus* (Ippolito et al., 2019; Novak et al., 2018) when BC was added with lime and F. Addition of BS to lime plus BC-amended soils showed an increase in shoot biomass and differences in nutrient availability and uptake (Trippe et al., 2021).

Two studies have addressed the impact of the addition of LSM to various combinations of tailing amendments. Trippe et al. (2021) showed that LSM addition resulted in changes in the rhizosphere but not in the bulk soil microbial community of blue wildrye. Frewert et al. (2022) showed that inoculation of BC-amended tailings with uncontaminated soils increased mycorrhizal colonization of both blue wildrye and Douglas fir (*Pseudotsuga menziesii* [Mirb.] Franco).

While the type of amendments used and results varied (e.g., from detrimental to beneficial effects of BC), these previous Formosa Mine tailings studies indicated the potential to successfully amend the Formosa tailings to support plant establishment and growth. They also indicated that beyond the necessity of lime to reduce tailing pH, the effects of other amendments, especially BC, on plant growth were mixed. Thus, further research using multiple combinations of remedial amendments is needed to better understand their impacts on the growth of native vegetation.

To develop specific remedial amendment mixtures to optimize the establishment, survival, and growth of native plants for revegetation of the Formosa Mine site and other sites, two greenhouse studies were conducted to address the hypothesis that lime, BS or F, BC, and LSM amendments reduce the constraints of tailings at the Formosa Mine and promote the establishment, survival, and growth of Douglas fir, a Formosa area native plant species. We included multiple levels of the different amendments to identify the best combination for amending the tailings found at the Formosa Mine to develop a stabilizing plant cover.

## 2 ∣ MATERIALS AND METHODS

### 2.1 ∣ Tailings and amendments

Bulk mine tailings for greenhouse experiments were obtained from the top 15 cm of mine tailings across the upper slopes of the Formosa Mine site. Tailings were passed through a 6-mm sieve in the field (Novak et al., 2018), followed by a 2-mm sieve in the laboratory. Amendments were added as % total weight, except for LSM. Lime was added as CaCO_3_ (Microna Agricultural Lime; Columbia River Carbonates) based on preliminary studies evaluating pH with different lime levels in the tailings over time (Text S1). BS were obtained from the Wastewater Treatment Plant, Myrtle Creek, Oregon, US. As a contrast to BS as a nutrient source in the second experiment, chemical fertilizer (F) was added as NH_4_NO_3_ (1.053 g), (NH_4_)_2_HPO_4_ (1.010 g), and KCl (0.168 g) in each container, for an equivalent 2% by weight treatment and adjusted proportionally for other F treatments. A gasified, high-temperature (1400°C) BC of mixed softwood wood waste from Douglas fir (*P. menziesii* [Mirb.] Franco), incense cedar (*Calocedrus decurrens* [Torr.] Florin), white fir (*Abies concolor* [Gord. & Glend.] Lindl. Ex. Hildebr), and sugar pine (*Pinus lambertiana* Douglas) was obtained from Cal Forest Nurseries. Portions of the material moved through the gasifier at lower temperatures (~1100–1200°C), with the exit gas temperature typically between 850 and 900°C. The appropriate proportions of tailings and amendments were weighed out and mixed for 10 min using a small cement mixer and placed into large tubs to provide a sufficient batch of growth media for each treatment.

To prepare LSM, soil from 0–7 cm below the organic layer (Oa horizon) was collected from an undisturbed forested area (latitude 42.8523760, longitude −123.3756600) near the Formosa Mine site, sieved through a 640-mm mesh, and stored at 4°C until inoculation. Sieved soil (1 kg) was added to an 11.36-L glass carboy that was filled with 10 L of M9 minimal salt media (Miller, 1972), supplemented with 0.1% glucose and 0.5% yeast extract. The carboy was capped with a twin bubble airlock and carboy bung and incubated at room temperature without shaking. After 30 days, LSM was harvested by filtering the culture through eight layers of cheesecloth, changing the cheesecloth when necessary, and aliquoting into 50-mL conical tubes (Thermo Scientific). LSM was pelleted by centrifugation for 30 min at 4000 rpm and 22°C in a 5810R Eppendorf centrifuge. Pellets from three tubes were combined, washed twice with 50 mL E-Pure H_2_O (Barnstead, Thermo Scientific), resuspended in 50 mL of E-Pure H_2_O (Barnstead, Thermo Scientific), and left to equilibrate overnight at room temperature. One tube of concentrated LSM was decanted onto the top of each tree container of the appropriate treatment. Treatments not receiving LSM were watered with an equal amount of water (50 mL).

### 2.2 ∣ Plant culture and measurements

All plants were grown in black plastic tubular containers (6.35 cm diameter × 24.77 cm high, 0.785 L volume). Black weed cloth was placed at the base of each container to prevent loss of amended tailings. Containers were held in place with black plastic honeycomb holders with a plastic cup under the container to collect and hold drainage water. Key plant culture dates for each experiment are shown in Table S1.

For Experiment 1, Douglas fir seedlings (seed source from foothills of Cascade mountains, Darrington, Snohomish County, Washington, elevation is 0–305 m) were obtained from Fir Run Nursery. Seeds were stratified in January 2016, sown in February, grown in the greenhouse until December or January 2017, and then put into cold storage at 1°C at the growers. Upon arriving in Corvallis, the seedings were kept at 4°C prior to transplanting on May 5, 2017. Seedlings were randomly selected from a population that was culled of those that were overly large or small. A small amount of tailings or amended tailings was put on the weed barrier cloth and the seedling was held in the container, while the remaining tailings and amendments for a specific treatment were added to the container. Containers were tapped on a counter to settle the tailings mix and provide headspace for watering. Twenty and 200 mL of water was added to the top and cup below the container, respectively.

For Experiment 2, 1-year-old Douglas fir seedlings (different seed source from Experiment 1) from close to the Formosa Mine near Silver Butte in Douglas County, Oregon) were obtained from Cal-Forest Nurseries. The seeds were sown in March 2017 in a mixture of 80% sphagnum peat and 20% fir sawdust in 15.2 cm deep × 2.5 cm diameter containers with liquid feed NPK fertilizer and micronutrients, and grown in a greenhouse until the plugs were removed on 26 September, 2017. The seedlings were kept in a cold room at 4°C for 24 days prior to planting on October 20, 2017. Seedling selection and planting were performed as described in Experiment 1. The containers were weighed and then increments of 50 mL were added if there was enough room at the top of the container. Water addition was repeated until breakthrough (~200 mL total).

For both experiments, plants were grown in a greenhouse with a target night temperature of 10°C (22:00–07:00 Pacific Standard Time or PST) and day temperature was 20°C (07:00–22:00 PST) for an 8/16-h light/dark period. For Experiment 2, during the fall and winter, supplemental blue, red, and white light were provided with light-emitting diode (LED) lights during the 16-h day period in order to stimulate bud break and off-season plant growth. Table S1 indicates the average environmental conditions for each experiment.

Regular supplemental watering was with reverse osmosis (RO) water on an individual container basis to maintain optimum tailings moisture. For Experiment 1, water was added to a 5-cm line from the bottom when the cup was empty; this was later adjusted to 1 cm. For Experiment 2, containers were watered twice per week and a cumulative measure of water use was determined. Containers were weighed and the amount of water to be added = (100 − [current wet weight − initial dry weight]). If the current wet weight of the container was heavier than the original dry weight + 100 g, no water was added. Water use by container per day (mL·day^−1^) was determined by summing water additions and dividing by the number of days. This represented water loss from transpiration, evaporation, and any water remaining in the cup at the end of the study for both live and dead trees.

Douglas fir pretreatment stem height was measured from the edge of the container to the top of the needles held stretched above the terminal bud. At the final harvest, tree survival was recorded and shoots were cut off from the root systems. Shoots were divided into preexisting (1-year-old at planting) needles and stems, and new growth. New needles and stems were combined for Experiment 1 but were separated for Experiment 2. As feasible, senesced needles on the top of the container were collected and included with the previous year’s needles. In Experiment 2, some needles were lost due to falling off the plant away from the container and from handling. For Experiment 1, root systems were immediately harvested by removing them from the tubes and washing away the tailings and amendments. For Experiment 2, after the shoots were removed, the containers with the roots were kept in the greenhouse and leached with RO water (described later). The containers were kept at 4°C until roots were harvested. Plant shoot and root materials were dried at 60°C for at least 3 days prior to weighing. Root data may be analyzed in a future paper along with LSM data.

For Experiment 2, Douglas fir new growth needles were analyzed for total Al, Ca, Cu, Fe, K, Mg, Mn, S, P, and Zn concentrations. Dry needles were cut into small pieces and digested using automated block digestors according to EPA method 3050B (EPA, 1996). The digestate was analyzed for total elemental concentrations with an Agilent 5110 ICP-OES by the USDA Agricultural Research Service (USDA, ARS).

### 2.3 ∣ Tailings, BS, BC, and leachate chemical measurements

Table 1 reports the chemical characteristics of the tailings, BS, and BC. For tailings and BS, three samples were evaluated for pH, total N, and total K, P, Ca, Cu, Fe, Mg, Mn, Zn, NO_3_-N, and NH_4_-N by the Oregon State University, Central Analytical Laboratory (OSU, CAL; https://cropandsoil.oregonstate.edu/shl/methods-and-equipment). Tailings and BS Al and Na (three samples) were extracted with Mehlich 3 and analyzed using a Varian ICP-OES (USDA, ARS). Tailings EC and S were measured on leachate from 10 tailings-only tubes at the end of Experiment 2 by the EPA, Pacific Ecological Systems Division (EPA, PESD) and USDA, ARS, respectively. Tailings EC was measured using a 2:1 MilliQ deionized water to tailings ratio (v/v), with an Amber Science Electrical Conductivity Meter (Model 4083), and S was measured as SO_4_-S with a Dionex Ion Chromatograph.

For BS, EC and pH were measured for four samples; for BC, EC and pH were measured for three samples by EPA, PESD as for tailings EC, but using a Sartorius PT-15 for pH as well as the Amber Science Electrical Conductivity Meter for EC (Model 4083), with EC measurements being made before pH measurements. For BS, the pH also was measured on three additional samples using a Hanna HI 764100 electrode by OSU CAL, resulting in a total of seven samples analyzed for pH.

For coarse BC, total N concentration was measured for one sample (EPA, PESD), using continuous-flow isotope ratio mass spectrometry (CF-IRMS, Model: ISOPRIME 100; Elementar Americas Inc.) coupled to an elemental analyzer inlet (Model: Vario ISOTOPE Cube). Three samples were analyzed for total K, P, Al, Cu, Fe, Mg, Mn, Na, and Zn at Bureau Veritas Commodities Canada Ltd. Samples were processed by Aqua Regia digestion and analyzed by Inductively Coupled Plasma Mass Spectrometry (ICP-MS). The procedure described in Olszyk et al. (2018) was used, except the analysis was with an Elan 9000 ICP-MS (Perkin Elmer).

At the end of Experiment 1, pH and EC were measured for the different tailings and amendments in each container after the roots had been removed, using the same procedure as for BS. At the end of Experiment 2, tailings leachate was obtained after the shoot harvest. Containers with intact root systems were individually weighed and placed in racks in the greenhouse with a clear plastic cup beneath each container to collect leachate. RO water was added to saturation (~100 mL), with an additional 250 mL of water used to flush the tailings and drain the leachate into the cup. Leachate was collected over 4 days, with most of the sample collected within 24 h. Leachate was transferred into clean plastic bottles and held at 4°C. The pH and EC were measured directly on the leachate solution using the same procedure as for BS, but without dilution. For total elemental concentrations, the leachate was passed through a 0.45-μm nylon filter and analyzed with an Agilent 5110 ICP-OES by the USDA, ARS.

### 2.4 ∣ Experimental design and statistical analysis

Detailed information concerning the statistical analysis procedures is provided in Text S2. Briefly, transformations in the Box–Cox power family of transformations were used if necessary for response parameters to help satisfy normality requirements. During analysis of the multiple treatment effects, model selection was performed using stepwise regression where in each step a predictor is considered for inclusion or exclusion based on acceptance criterion established prior to model fitting. The Bayesian information criterion (BIC), a criterion based on finding models with lower BICs, was used to help compare models and select predictors. Tree height (preheight) at planting was tested as a covariate in most analyses. Tree survival was analyzed by logistic regression and Chi-squared tests.

**FIGURE 1:**
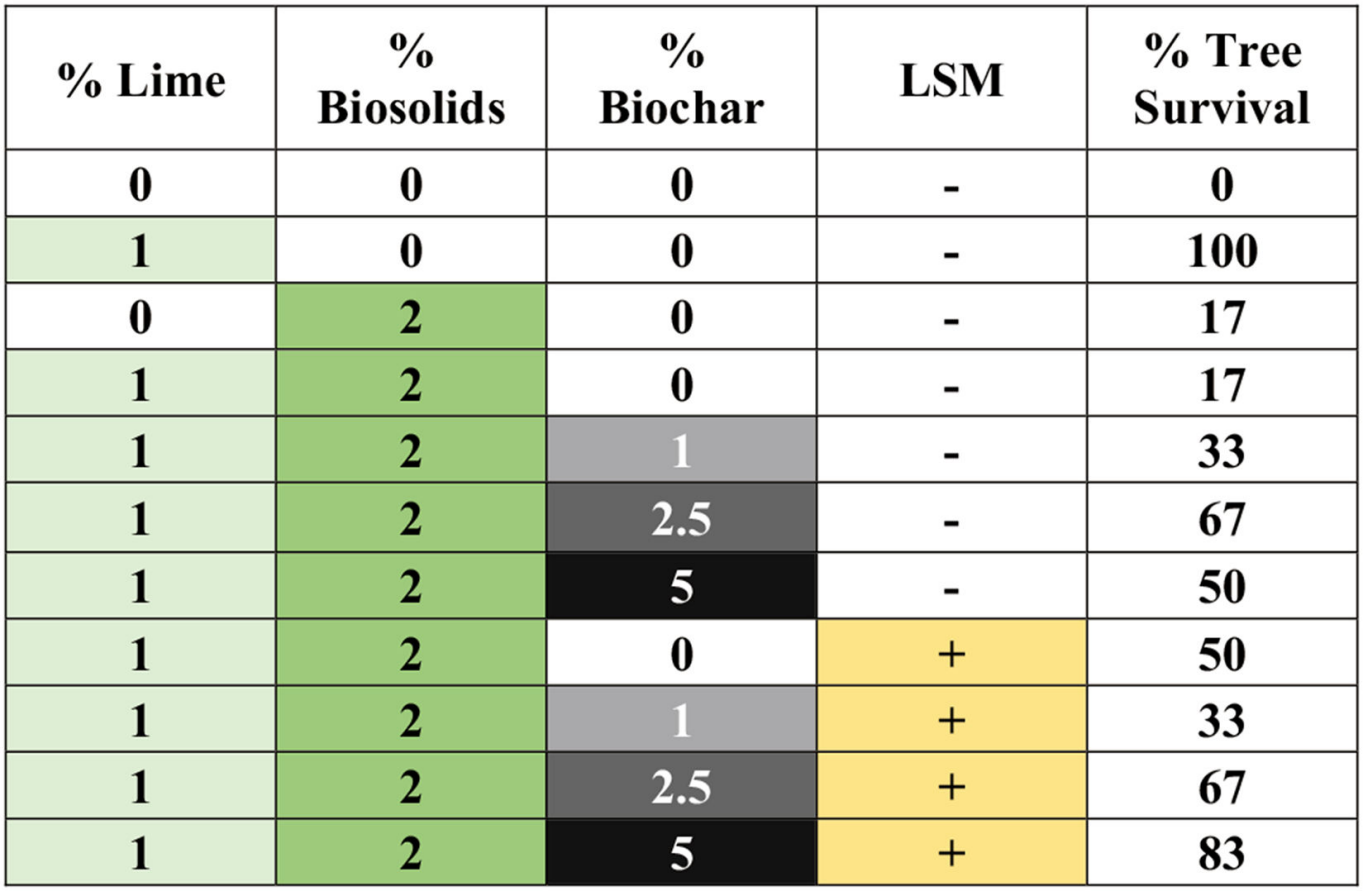
Matrix of effects of amendment treatments to Formosa Mine tailings and Douglas fir survival in Experiment 1. Higher intensity color indicates higher level of amendment (white = no amendment). LSM, locally sourced microbes. The % tree survival is for *N* = 6.

Experiment 1 was an initial exploratory study to determine if a limited set of amendments allowed for plant survival and growth in Formosa Mine tailings. There were 11 treatments: tailings alone, tailings plus L (1%) or BS (2%) alone, tailings plus L plus BS and 0%, 1%, 2.5%, or 5% BC, and tailings plus L plus BS plus LSM and 0%, 1%, 2.5%, or 5% BC (Figure 1). There were six replicate containers per treatment in blocks, with replicate containers for all treatments randomly located across all plastic holders on the greenhouse bench. This experiment addressed five questions: (1) Does 1% lime alone affect plant survival? (2) Does 2% BS alone affect survival? (3) Does the addition of BS affect the response to lime? (4) Does the addition of BC affect the response to BS and lime? (5) Do LSM affect the response to the mixture of lime, BS, and BC? All containers were used in the analysis of tailing pH, EC, and tree survival. Data for new shoot weight for live trees (dead trees and live trees without new growth not included) are shown in the Supporting Information for reference purposes only. Data for pH and EC were subject to regression analysis to determine the best fit for a change in the parameter with a change in BC level from 0% to 5% considering LSM (Figure S1).

**FIGURE 2:**
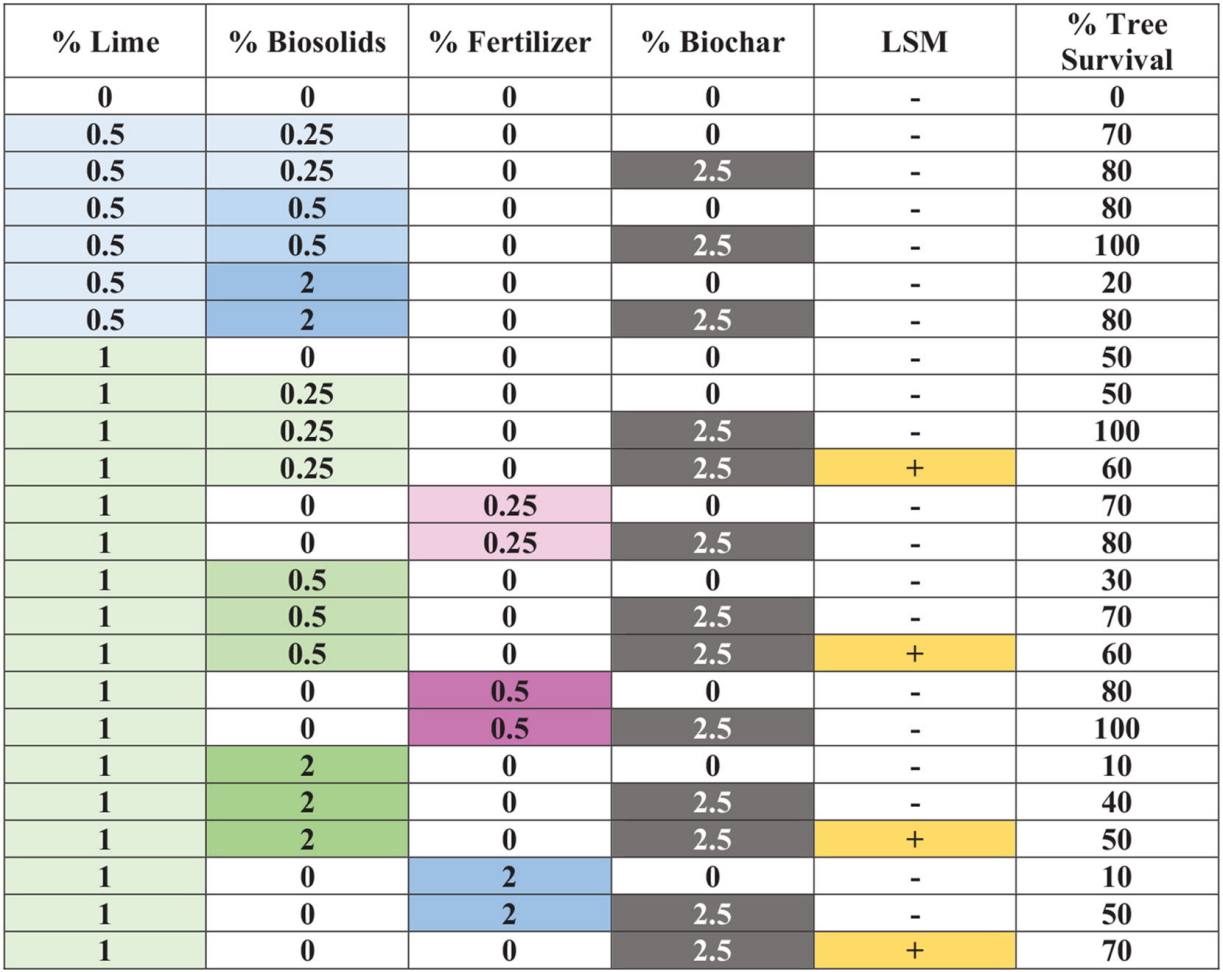
Matrix of effects of amendment treatments to Formosa Mine tailings and Douglas fir survival in Experiment 2. Higher intensity color indicates higher level of amendment (white = no amendment). LSM, locally sourced microbes. The % tree survival is for *N* = 10.

Experiment 2 was designed to determine how different levels of lime, type of nutrient source (BS or F as an alternative to BS), level of nutrient source, BC, and LSM affect Douglas fir seedling growth independently and their interactions, in order to better suggest amendment combinations for the field. The treatments were distributed in a randomized complete block design where Douglas fir seedlings were randomly assigned to one of 24 treatments within each of the 10 blocks. The blocks were arranged in two groups of five on a greenhouse bench. The 24 treatments included tailings alone; tailings plus 1% lime; tailings plus 0.5% or 1% lime and 0.25%, 0.5%, or 2% BS, all with or without BC; tailings plus 1% lime and 0.25%, 0.5%, or 2% F, all with or without BC; and tailings plus 1% lime and 0%, 0.25%, 0.5%, or 2% BS, BC, and LSM (Figure 2). The statistical analysis addressed four questions of interest: (1) What is the effect of 1% lime lone versus the unamended tailings on a response parameter? (2) Is there a difference in the effect of 0.5% or 1% lime on a response parameter with different levels of BS (0.25%, 0.5%, and 2%) with or without 2.5% BC? (3) Is there a difference in the effect of BC on the response parameter for the different sources of nutrients (BS or fertilizer) and different levels of nutrients, all at 1% lime? (4) Is there a difference in the effect of presence or absence of LSM on the response parameter when BS is present at three different levels, all at 1% lime and 2.5% BC?

All containers with live or dead plants were used in the analyses in Experiment 2 in order to determine the impacts of treatments on leachate pH, EC, and chemistry. Only live trees and treatments with at least one live tree per treatment were analyzed for new needle growth and elements. If a tree was alive, but had no new growth, a 0 was used for new needle dry weight. The resulting number of replicate trees per treatment for new needle dry weight and elements is shown in Table S2.

The use of four separate models for Experiment 2 resulted in one test for lime versus unamended tailings, one test for lime level, one test for nutrient source type, one test for LSM presence, two tests for BC presence, and three tests for nutrient level (Tables S4 and S5). For BC, at least one of the tests had to be significant and for nutrient level at least two of the tests generally had to be significant for BC or nutrient level to be considered overall significant; however, the significance of the main effect may be qualified by interactions.

The comparison of 1% lime alone and tailings without amendments was performed using SAS (SAS Institute, SAS version 9.4, SAS/STAT 15.1 Proc Logistic with Contrast option). The other analyses were performed using R statistical software, version 3.5.0 (R Core Team, 2018) (Text S2). Boxplots (the majority graphed in SigmaPlot Version 15, with a few in SAS [SAS version 9.4, Proc Sgplot, SAS]) were used to visualize most parameters except for tree survival where a matrix with different colors for treatments was used.

Pearson correlation coefficients and a heat map were used to visualize relationships among survival, new needle dry weight, new needle nutrients, and leachate chemistry in Experiment 2. Different sizes and colors of circles indicated the direction and significance of correlations. The heat map was based on a correlation matrix of Pearson’s *r* and approximated *p*-values computed in R (3.6.1) using rcorr from the Hmisc (4.4.0) package. A correlation matrix was plotted showing only those correlations with no effect at *p* > 0.05 with corrplot (0.84).

## 3 ∣ RESULTS

### 3.1 ∣ Experiment 1: Douglas fir seedling survival

For Experiment 1, the results of the statistical analysis are summarized in Table 2 and the *p* values from the ANCOVAs and ANOVAs are shown in Table S3. In the unamended tailings, all Douglas fir seedlings rapidly died after planting, likely due to the low pH (Figures 1 and S1a). The addition of lime alone allowed all Douglas seedlings to survive and grow (Table 2; Figure 1). In contrast, amending the tailings with BS alone or BS and lime resulted in very low tree survival (only one tree; Figure 1). Seedling survival tended to increase with the addition of BC (*p* = 0.095) (Figure 1). There was no overall significant effect of LSM; however, survival was higher with LSM than without LSM at the lowest (0%) and highest (5%) BC application rates. While the data for live trees with new growth were only for reference purposes and not analyzed statistically, the new shoot growth tended to be greater with higher BC levels, especially at 5% (Figure S2).

### 3.2 ∣ Experiment 1: Tailings pH and EC

Amendments significantly affected tailings pH and EC (Table 1). Lime alone increased pH from 2.7 to 6.7 (Figure S1a) and tended to reduce EC from 2.266 to 1.789 mS·cm^−1^, even though the effect of lime alone was not statistically significant (*p* = 0.184; Figure S1b). The addition of BS alone slightly increased pH to 3.5, but increased EC to 3.339 mS·cm^−1^. In the lime-amended tailings, the addition of BS decreased pH slightly, but considerably increased EC. The addition of BC to the lime and BS increased pH and decreased EC compared with lime alone. The LSM treatment slightly, but significantly, increased the BC effect on pH but had no effect on EC. In Experiment 1, the survival of the seedlings was affected by a phytotoxic response to the high application rate of BS; therefore, we conducted a second experiment that examined variable rates of BS application and compared the application of BS to mineral F.

### 3.3 ∣ Experiment 2: Douglas fir seedling survival, new needle growth, and needle elements

The results of the statistical analysis for Experiment 2 are summarized in Table 3 and the *p* values from selected models are shown in Tables S4 and S5. Only the general effects of the LSM amendments on Douglas fir seedlings are discussed here. A detailed analysis of LSM on tailing characteristics and tailing microbial biomass and community characteristics is beyond the scope of this paper and will be discussed in a future publication. Douglas fir bud break and new shoot growth were uneven across all treatments due to forcing the seedlings to grow over the winter in a greenhouse. Across all 141 trees that survived to harvest, only 47 had terminal bud break, while 139 had terminal or axillary bud break. Of the 100 trees that died, only six had buds that had broken (one terminal bud had broken) before the trees died.

The addition of 1% lime alone increased the survival of Douglas fir seedlings compared with the unamended treatment even though the difference was not statistically significant (Table 3; Figure 2). However, survival in Experiment 2 was only half the 100% survival observed in Experiment 1 with 1% lime alone added to the tailings (Figure 1). There was lower survival with 1% than with 0.5% lime primarily due to increased toxicity of higher BS levels with higher tailing pH. There was slightly lower tree survival across all BS than F treatments. Amending the tailings with the higher level of lime (1%) and the highest level of nutrient (2% of either BS or F) and no BC or LSM was most detrimental to the trees, with only one tree per treatment surviving (10%). In contrast, there was 30%–80% survival with 1% lime and lower levels of 0.25% or 0.5% nutrient and no BC across LSM levels (Figure 2). Adding 2.5% BC to the amendments increased tree survival compared with each corresponding non-BC treatment; 100% survival was achieved with BC and 0.5% lime and 0.5% BS, and BC plus 1% lime and 0.25% BS or 0.5% F (all without LSM). The addition of LSM had no statistical effect on survival.

**FIGURE 3:**
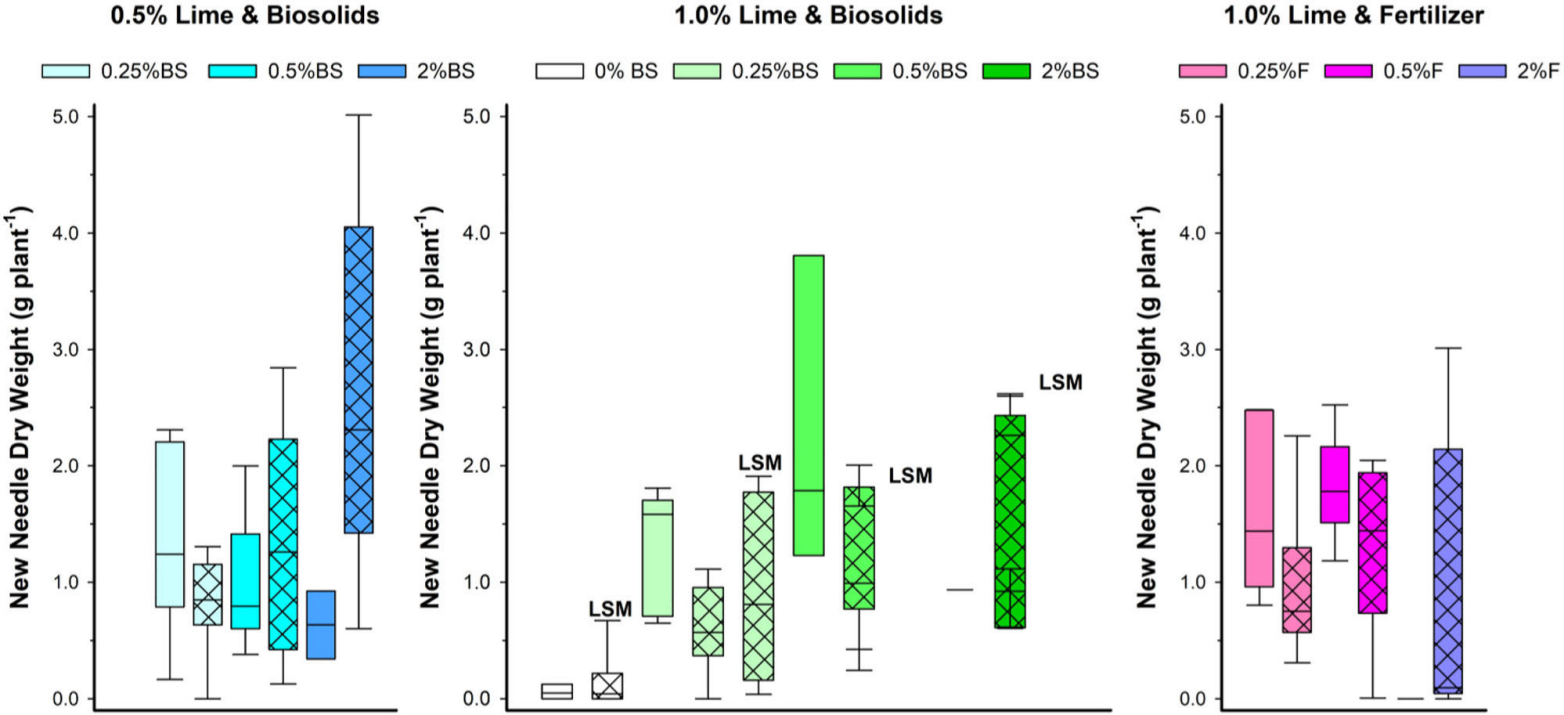
Boxplots for effects of amendment treatments to Formosa Mine tailings on Douglas fir new needle dry weight in Experiment 2. Dead plants not included; live plants without new needles (0 weight) were included. BS, biosolids; F, fertilizer; LSM, locally sourced microbes. Black vertical line in bar is median; cross hatch in bar is 2.5% BC; and the darker the color, the higher the BS or F level. *N* = 0–10 depending on treatment (see Table S2).

**FIGURE 4:**
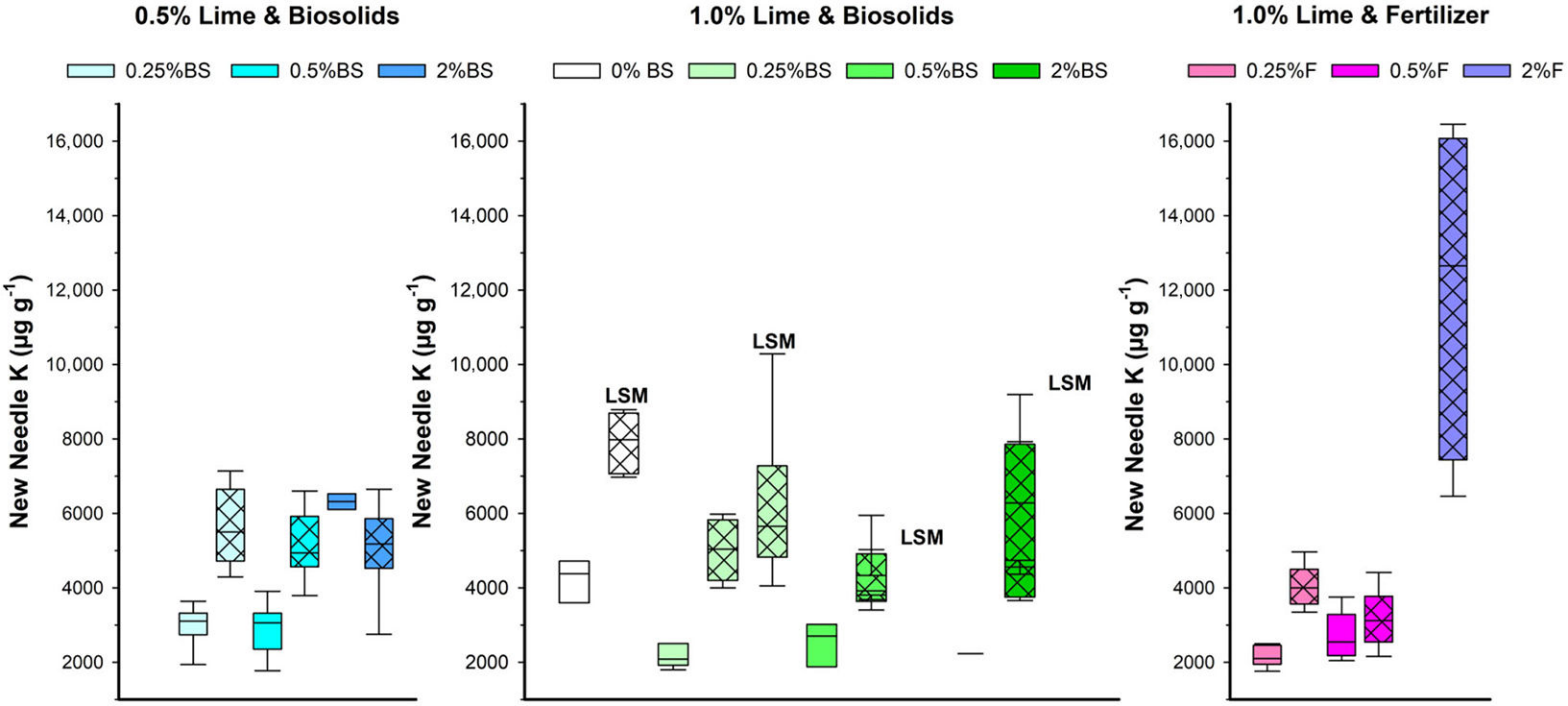
Boxplots for effects of amendment treatments to Formosa Mine tailings on Douglas fir new needle K in Experiment 2. BS, biosolids; F, fertilizer; LSM, locally sourced microbes. Black vertical line in bar is median; cross hatch in bar is 2.5% BC; and the darker the color, the higher the BS or F level. *N* = 0–10 depending on treatment (see Table S2).

Due to the large number of dead trees, statistically determining the effects of the treatments on new needle dry weight was difficult, with no factor consistently significant across all analyses (Tables 3 and S4; Figure 3). There were some significant effects and interactions based on the statistical analysis, but with considerable variability among treatments. BC reduced new needle dry weight across nutrient sources and levels, but there was no BC effect when only lime and BS were considered. Elemental concentrations in the new needles reflected available nutrient sources in the amended tailings. For the essential nutrient K, the needle concentration increased when BC was added with other amendments (Table 3; Figure 4). There were many interactions between factors for needle K as indicated in Table 3. In contrast to K, BC reduced the new needle concentration of another essential element, Ca (Table 3; Figure S3). The addition of a higher concentration of plant nutrients significantly increased Ca in new needles, regardless of source. Neither lime nor LSM influenced K and Ca in needles. Although this response was variable among treatments, the new needle Cu results suggest beneficial effects of lime and BC in reducing plant uptake of the potentially toxic heavy metal Cu (Table 3; Figure S4). Though there was a statistically significant difference in new needle Cu between nutrient sources, it was likely not meaningful (Table 3). The nutrient level and LSM had no effect on Cu concentrations in needles.

### 3.4 ∣ Experiment 2: Tailings chemistry

Amendments had complex, often interacting effects on leachate pH, EC, and elemental composition. Detailed presentation and analysis of the results for the tailings responses for most leachate parameters are beyond the scope of this paper; however, the general results from the statistical analysis for key tailings leachate parameters are shown in Tables 3 and S5.

**FIGURE 5:**
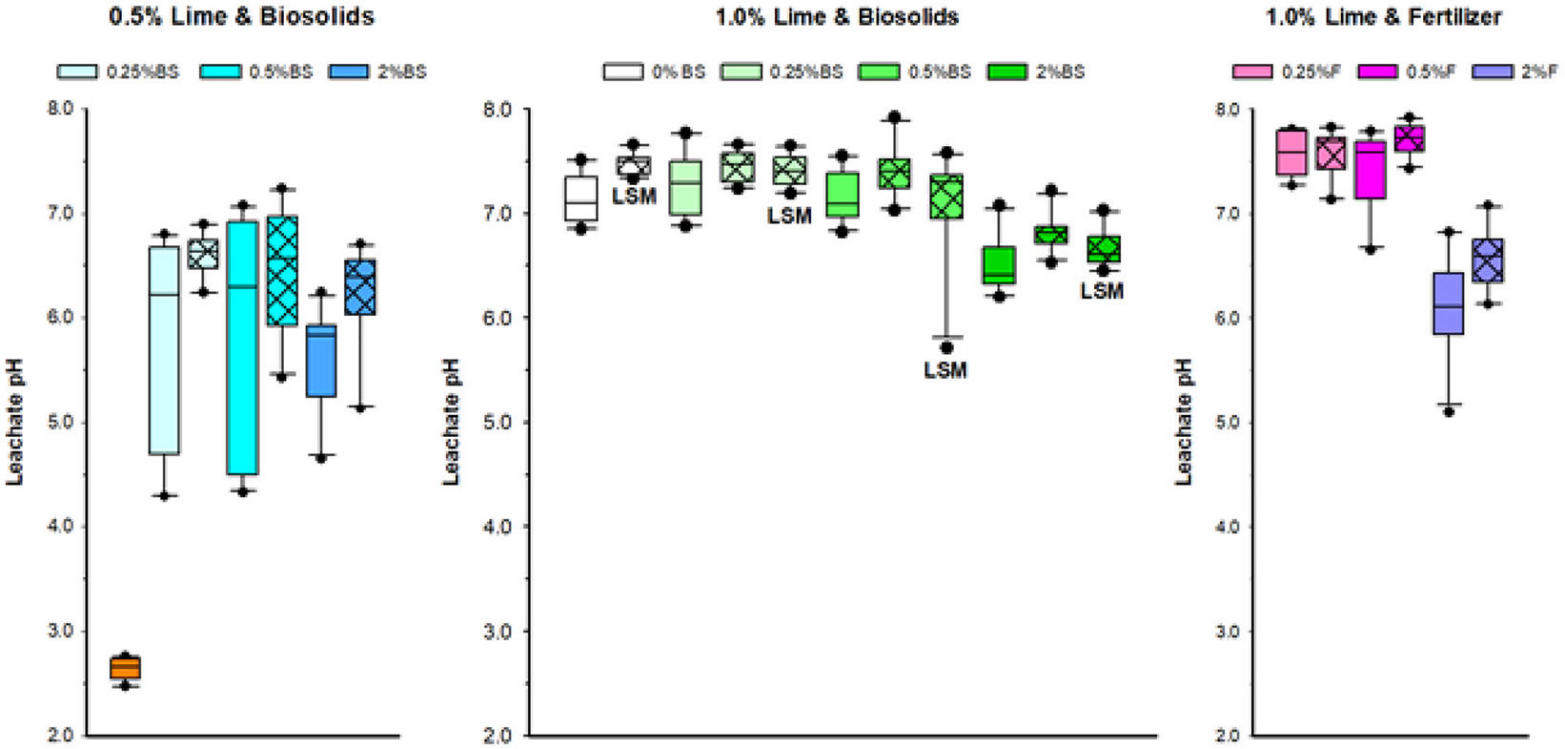
Boxplots of effects of amendment treatments to Formosa Mine tailings on leachate pH in Experiment 2. BS, biosolids; F, fertilizer; LSM, locally sourced microbes. Black vertical line in bar is median; cross hatch in bar is 2.5% BC; the darker the color, the higher the BS or F level; and orange bar in the far lower left of 0.5% lime and BS panel is the tailings without amendments. *N* = 10 per treatment.

As in Experiment 1, adding lime dramatically increased the pH of the leachate in Experiment 2 (Table 3; Figure 5). The pH of the leachate increased from an average of 2.6 for the tailings alone to just above 6.0 across all tailing treatments with 0.5% lime. Increasing the lime amendment from 0.5% to 1% further increased the pH of all treatments. Other amendments had less effect on pH than lime. In treatments with 0.5% or 1% lime, the highest level of nutrients (2%) decreased pH compared with other treatments, regardless of the addition of BC or LSM. Nutrient sources had mixed effects on leachate pH. The addition of BC increased pH. There was a small, but significant, decrease in pH with the addition of LSM.

In contrast to the clear effects on tailings EC in Experiment 1, the many factors in Experiment 2 resulted in complex interactions for leachate EC (Table 3; Figure S5). Adding 1% lime alone decreased EC from 3.929 to 2.128 mS·cm^−1^. Across all other amendments, increasing lime from 0.5% to 1.0% did not have a significant effect on EC. For the 1% lime treatments, increasing levels of nutrients (BS or F) from 0.25% to 2% significantly increased leachate EC from 3.211 to 10.665 mS·cm^−1^, respectively, across BC and LSM treatments. The use of BS as a source of nutrients resulted in a significantly (though small) lower EC than the use of F. Adding BC increased EC, but only at the lowest nutrient level of BS or F, while adding LSM lowered EC only at the highest BS level. The highest EC overall was with 1% lime, 2% F, and no BC or LSM (12.597 mS·cm^−1^).

At the end of Experiment 2, leachate concentrations of heavy metals were nondetectable or very low in nearly all treatments containing lime compared with the unamended tailings. For example, concentrations of leachate Al and Cu were essentially 0 (maximum values of only 0.4 μg·mL^−1^ for each element) for all lime treatments versus 202 μg·mL^−1^ for Al and 25 μg·mL^−1^ for Cu, in the tailings leachate (data not shown). A low concentration of Zn (2.3 μg·mL^−1^) was detected at low lime (0.5%) and BS (0.25%) when no BC was added, which was further reduced to 0.3 μg·mL^−1^ with the addition of BC, indicating significant BC and BC × nutrient effects (Table S5; Figure S6). Across amended treatments, leachate Zn concentrations were much lower than that for the unamended tailings (average of 24.1 μg·mL^−1^).

Increasing the nutrient level with BS or F, or adding BC increased leachate K, especially at the highest nutrient level, while adding lime or LSM did not increase leachate K (Table S5; Figure S7). There were interactions among amendments, such as a higher leachate K with 2% BS or F, significant only when BC was not present. A higher BS or F amendment rate also tended to increase the leachate concentrations of most other available nutrients (Ca, Mg, Mn, Na, and P; data not shown), with some other individual amendment effects and a number of interactions among amendments. Increases in concentrations of all elements were likely associated with the increases in leachate EC with higher BS or F levels (Figure S5).

**FIGURE 6:**
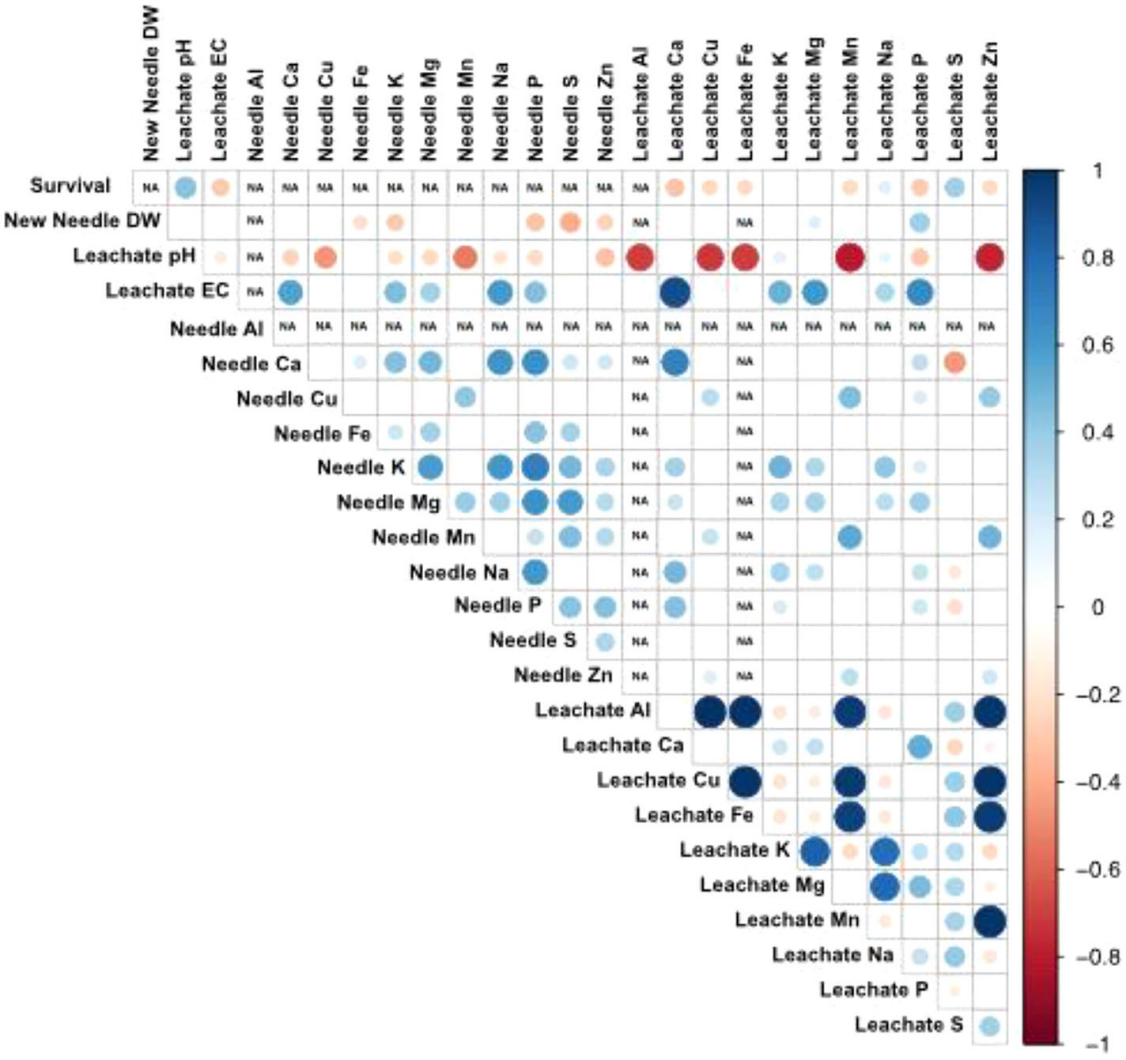
Heat map of Pearson correlation coefficients (PCC) for Douglas fir plant parameters (survival, new needle dry weight, new needle elements), and tailing leachate chemistry across all treatments in Experiment 2 to indicate general relationships among parameters. Circles are for comparisons with significant PCC with *p* value no effect at > 0.05, with larger circles indicating a higher PCC value; the range of colors corresponds to the direction of the PCC (blue for positive and red for negative). The *N* = 240 for plant survival and leachate chemistry, *N* = 141 for new needle dry weight, and *N* = 131 for needle elements. There were no correlations between survival and new needle weight or elements as only surviving trees were included for new needle parameters. There were no correlations between leachate Fe and needle elements because all leachate Fe concentrations were 0 for trees with new needles. Correlations were not used between needle Al and other parameters due to the low number (7) of non-0 needle Al values, and no correlations used between leachate Al and tree parameters due to the low number (6) of non-0 leachate Al values.

The correlation analysis showed significant relationships among tree survival and tailings chemistry parameters, among new needle dry weight, new needle elements and tailings chemistry parameters; and among tailings chemical parameters across treatments (Figure 6). For example, leachate pH was positively correlated with tree survival, but negatively correlated with new needle elements and the leachate elements. In contrast, leachate EC was negatively correlated to tree survival, but positively correlated with most new needles and the leachate elements. For a specific element, needle concentrations were, in general, positively related to leachate concentrations. The main exception was that needle Ca, and to a lesser extent needle Na and P, was negatively related to leachate S. Correlations among needle elements were positive. The direction and size of correlations among leachate elements varied by the element, with the strongest positive correlations among tailing metals—Al, Cu, Fe, Mn, and Zn.

## 4 ∣ DISCUSSION

### 4.1 ∣ Importance of specific amendments

Both experiments indicated that a mix of amendments is useful for altering tailings chemistry and facilitating plant growth, as suggested in previous studies of Formosa Mine tailings with more limited sets of amendments (Frewert et al., 2022; Ippolito et al., 2019; Novak et al., 2018; Phillips et al., 2016; Trippe et al., 2021). However, due to the extremely low pH of the tailings, some plants did not survive or grow even with amendments that should have resulted in plant survival. This result may be related to the fact that some time is required for amendments to react with the tailings and favorably alter their chemistry before planting. In the future, amendments could be mixed with the tailings and allowed to equilibrate for some time before planting seedlings. Each amendment played a specific role in the survival and growth of trees, which must be considered in designing the optimum mixture to be used in a field study.

#### 4.1.1 ∣ Lime

Lime was the most important factor for plant survival and growth across both experiments. This was expected due to the widespread recognition that lime is important to reduce acid mine drainage problems (Skousen, 2014), and the importance of lime to allow for plant growth in a previous study with the Formosa Mine tailings (Novak et al., 2018). Overall, the addition of lime resulted in less survival of Douglas fir seedlings in Experiment 2 (50%; Figure 3) than in Experiment 1 (100%; Figure 1a). The reason for this is unknown, but this may have been related to the poorer bud break associated with the off-season forced new growth in Experiment 2, which resulted in more variable and poorer tree growth in Experiment 2. Though the trees received supplemental lighting to extend the photoperiod and encourage bud break and growth, they may not have had the chilling required for normal bud break in Experiment 2 (Harrington & Gould, 2015).

Lime greatly increased tailings pH in both experiments (Figures S2 and 5) and reduced plant-available heavy metals (e.g., Zn; Figure S6). While 0.5% lime was sufficient to increase pH and make most heavy metals unavailable, 1% lime raised the pH the most and removed all heavy metals (as indicated for Zn in Figure S6). However, the higher pH with 1% lime may have also exacerbated phytotoxic effects caused by the highest level of plant nutrients (2%). This could be due to an associated increase in EC or increased volatilization of NH_4_ (Neina, 2019). These effects were minimized when the nutrient concentration in the amendments was reduced to 0.25% or 0.5%. Because no plant nutrients were added with lime alone and the Formosa tailings were low in nutrients (Table 1), the new growth with lime alone likely was supported by the reallocation of nutrients from the older needles to the new needles (Hawkings et al., 1998).

As both the increase in pH and associated decreases in leachate metals with the amendments (likely primarily due to lime) were correlated with increased plant survival (Figure 6), it is uncertain whether the initial low pH itself or metal toxicity in the unamended tailings resulted in plant death. The metals of primary concern in these tailings were Al, Cu, and Zn. There was a range of 450–2371 mg·kg^−1^ Al, 36–252 mg·kg^−1^ Cu, and 102–606 mg·kg^−1^ Zn in old needles of dead trees from the no-lime treatment (data not shown) indicating that toxicity of these elements could have contributed to their rapid death. According to Kabata-Pendias (2010), growth depression occurs at 15–20 mg·kg^−1^ Cu, and an upper toxic range for Zn is 100–500 mg·kg^−1^ in plant tissues. The initial site of Al toxicity symptoms in plants is the root system (Bojórquez-Quintal et al., 2017). Based on research with somatic plantlets and 1 mM AlCl_3_ in the medium (Amara et al., 2020), the level of Al associated with a growth reduction in Douglas fir was 63 mg·kg^−1^ dry weight in needles and much higher in roots at 330 mg·kg^−1^ dry weight. In fact, while roots and stems had reductions in dry weight with 1 mM AlCl_3_, the dry weight of the needles actually increased (Amara et al., 2020). Only 0–11 mg·kg^−1^ Al, 0–4 mg·kg^−1^ Cu, and 6–219 mg·kg^−1^ Zn were measured in new needles of live trees across all treatments in our study, suggesting that the low leachate concentrations of these elements with the amendments (especially lime) were associated with reduced plant uptake, likely benefiting tree survival.

#### 4.1.2 ∣ Nutrient source and level

Because adding essential nutrients is necessary for the long-term growth of plants, a convenient source is necessary for field remediation. In this study, the source (BS vs. F) had a slight effect on Douglas fir in these short-term experiments, with greater survival with F (*p* = 0.031 across all treatments; Tables 3 and S4). The nutrient source had only minor effects on tailings chemistry, with the F treatment producing a higher tailings EC. This higher EC was likely due to the solubility of this fertilizer designed to rapidly provide plant nutrients.

This study clearly indicated that applying excessive nutrients was detrimental to plant survival. In Experiment 1, where only a high level (2% by weight) of BS was used, the BS countered the beneficial effects of lime by reducing survival (Figure 1). In Experiment 2, both the highest BS and F reduced survival (Figure 3). The inhibition of survival with the addition of high nutrients to lime was likely related to the high EC (Figures S1b and S5), with a possible contribution for BS from the total N made available to the trees, especially the large pool of 3143 mg·kg^−1^ NH_3_ (Table 1). This concentration of NH_3_ may have phytotoxic effects (Esteban et al., 2016).

#### 4.1.3 ∣ Biochar

This study confirmed that BC may complement other amendments to establish vegetative cover at mining-impacted sites with sulfidic tailings as indicated by Sizmur et al. (2016). BC had beneficial effects on tailings chemistry in this study by enhancing the pH-increasing effect of lime, while likely providing additional benefits to plants. BC likely enhanced plant survival in both experiments, especially suggested in Experiment 1 where multiple levels of BC were used (Figure 1). In Experiment 2, enhanced plant survival with BC was indicated by the significantly more live trees with 2.5% versus 0% BC across different lime and BS or F levels (Table 3). Enhanced plant survival with BC also was suggested by 60%–100% survival across all BC treatments with 1 % lime and 0.255 or 0.5% BS or F compared with only 50% survival with 1% lime alone (Figure 2).

The optimal BC level based on survival in Experiment 1 maybe 2.5%, similar to the 2% BC that enhanced initial rye growth in the preliminary experiment with the Formosa tailings by Ippolito et al. (2019). However, the optimum level of BC depends on the presence of other amendments. For example, Phillips et al. (2016) indicated that, in the absence of lime, higher rates (at least 4%, likely 9%) of BC alone are necessary to foster plant growth by increasing the Formosa tailings pH and reducing the availability of heavy metals. BC generally has a lower bulk density than soil (less than half; Blanco-Canqui, 2017; Verheijen et al., 2019); therefore, a large volume of BC would need to be applied to achieve even 2.5% by weight over a large area of tailings, making BC logistically and financially challenging to apply and incorporate. These challenges may be overcome by adding BC or BC-based mixtures into furrows, rows, or planting holes to effectively lower the overall area requiring amendment. This method has been successfully used to apply BC directly to root zones (Sales et al., 2022; Schmidt et al., 2017); however, the long-term consequences of this approach are uncertain.

The effect of BC application rate is likely related to the BC’s physiochemical properties, which are defined by feedstock origin and production conditions (Ippolito et al., 2020), and the benefits those properties confer. For example, Novak et al. (2018) reported that the root biomass of blue wildrye grown in Formosa Mine tailings was affected by 1% BC, added to lime, and F mixtures; higher application rates did not affect growth. In that study, a miscanthus BC, pyrolyzed at 700°C, was used at a similar rate (2.5% by weight), as in our study. This suggests that in our study, additional benefits of BC were realized that directly impacted the ability of plants to survive and grow in mine tailings. The BC likely sorbed excess salts resulting in a decreased EC as shown in Experiment 1 (Figure S2b), thereby reducing tailings salinity as suggested by a previous soil study (Huang et al., 2019). BC alleviated some of the adverse effects of the high nutrient concentration (2% BS or F) on survival in experiment 2, as indicated by the greater survival with BC (Figure 3). The BC further reduced heavy metal concentrations beyond the effect of lime, as indicated by reduced Zn with 0.5% lime in Experiment 2 (Figure S6). BC was shown in several studies to decrease available heavy metals in soils (Ippolito et al., 2019; Phillips et al., 2016; Shi et al., 2022). In addition, the increase in needle K with BC (Figure 4) confirmed that BC also can provide certain essential plant nutrients, especially K (Wang et al., 2018).

In this study, tailings moisture was artificially maintained; therefore, the potential benefits of BC in increasing moisture retention/holding capacity (Novak et al., 2012) of the coarse-textured tailings may not have been observed. However, a supplemental experiment indicated that BC could increase the moisture-holding capacity of the mine tailings (Text S3), which would be important in field settings, especially for dry sites.

Overall, our study indicated the potential usefulness of BC for the restoration of abandoned mine tailings. The use of locally sourced wood-based BC may have benefits in terms of sustainable forest management, provided the right characteristics of BC are considered and concerns about possible contaminants in the BC are addressed (Rodriguez-Franco & Page-Dumroese, 2021).

#### 4.1.4 ∣ Locally sourced microbes

Locally sourced microbes had no significant effect on Douglas fir survival in either experiment. The suggestion of a possible increase in survival with LSM in Experiment 1 could indicate that LSM is responsible for mobilizing nutrients to improve growth (Qi et al., 2022), which warrants further investigation. In contrast, based on the limited tailings chemistry data in this paper, there were effects, though in opposing directions, of LSM on tailings pH in both experiments. A lack of plant effects was observed in earlier studies evaluating the effects of LSM or soil transplants on blue wildrye grown in amended Formosa tailings (Frewert et al., 2022; Trippe et al., 2021). However, all of these results were obtained from short-term studies; longer term studies may be required to observe the benefits and plant response to microbial amendments that occur over time. A detailed analysis of changes in the tailing chemistry and effects on microbial community composition and tree responses with LSM at the end of the experiment will be given in a future paper.

### 4.2 ∣ Usefulness of greenhouse studies

This study indicated that multi-month greenhouse studies with containers can provide information concerning the effects of amendments on the survival and growth of plants in amended mine tailings. Compared with field studies, container studies provide a relatively simple platform for evaluating a large number of amendment types and growth under standardized conditions to design amendment treatments for further investigation. In this study, a combination of 1% lime, 0.25% or 0.5% nutrient, and 2.5% BC appeared to produce optimum plant responses, and this suite of amendments is being used in a complimentary field study (Johnson et al., unpublished data). However, container studies such as those used in these experiments should be approached with caution as they may overestimate the effects of BC on plant responses compared with field studies (Jeffrey et al., 2011). Nonetheless, as suggested by Rieder et al. (2013), a combination of greenhouse container and field studies can inform restoration strategies at the Formosa Mine and at other sites. Because reclamation of abandoned mine land is a very complex process, over time the assessment of the reclaimed site is necessary to evaluate the success of reclamation, especially by focusing on the soil microflora community (Sheoran et al., 2010). To fully understand the potential for the use of BC for remediation on abandoned mines and heavy-metal-contaminated soils (Ghosh & Maiti, 2020, 2021; Wang et al., 2021), long-term, field-based studies with trees are needed.

## 5 ∣ CONCLUSIONS

These experiments clearly confirmed that lime is a critical amendment for growing plants in acidic mine tailings such as at the Formosa Mine. A source of nutrients such as BS or F also is necessary for long-term plant survival and growth; however, their effects on tailings EC must be carefully considered, and a too high concentration of nutrients can adversely affect tree survival. Amendment with BC caused additional changes in tailing chemistry that may further enhance tree survival beyond the effects of lime. Based on concentrations used in these experiments, a combination of amendments with 1% lime, 0.25% or 0.5% BS or F, and 2.5% BC by weight is suggested for further study. These results will be useful to design field experiments to determine amendments for the establishment and growth of Douglas fir at the Formosa Mine site and will be applicable to revegetation plans for other abandoned mine sites.

## Supplementary Material

Supplement1

## Figures and Tables

**TABLE 1:** The pH, electrical conductivity (EC), and elemental concentrations for the Formosa Mine tailings, biosolids (BS), and biochar (BC).

Material	pH	EC	N	K	P	Al	Ca	Cu	Fe	Mg	Mn	Na	S	Zn
		mS·cm^−1^	—————————————mg·kg^−1^—————————————											
Tailings^a^	2.5	3.929	43	18	5	823	1886	38	536	53	19	24	226	44
Biosolids^b^	5.8	3.166	75,620	3982	8292	141	3302	9	576	3211	84	541	–	95
Biochar^c^	9.8	2.909	2189	94,000	8533	5467	161,133	112	21,333	53,100	6910	1110	1367	99

*Note*: As a result of different analysis methodologies, tailings and BS elements are not directly comparable to BC elements.

a Mine tailing results for pH, total N, and K, P, Al, Ca, Cu, Fe, Mg, Mn, Na and Zn for *N* = 3 samples. In addition to the data in the table, NO_3_-N below quantifiable limits and NH_4_-N = 3 mg·kg^−1^, *N* = 3. Results for EC and S as SO_4_-S, *N* = 10.

b Biosolids results for pH, *N* = 7; for EC *N* = 4; and elements, *N* = 3. In addition to the data in the table, NO_3_-N 6.9 = mg·kg^−1^ and NH_4_-N = 3143 mg·kg^−1^ for *N* = 3. No data are available for biosolids S.

c Biochar results for total N, *N* = 1, and for pH, EC, and other elements, *N* = 3.

**TABLE 2:** Summary of statistical analysis for effects of lime, biosolids (BS), biochar (BC), and locally sourced microbes (LSM) on Douglas fir seedling survival (measured as 1 = live and 0 = dead) and tailing chemistry for plants grown in the Formosa Mine tailings in experiment one.^a^

Parameter	*n*	Main effects^a^				
		Lime (1% vs. 0%)	BS (2% vs. 0%)	BS + lime (vs. lime)^b^	BC (0%, 1%, 2.5%, 5%)^c^	LSM (yes vs. no)^d^
pH	66	+	+	−	+^e^	+
EC	66	ns	+	+	−	ns
Survival	66	+	+^f^	−	ns^g^	ns

a “+” indicates a significant increase in the parameter from the first parenthetical variable to the second, “−” significant decrease, and “ns” no effect at *p* > 0.05 according to analysis of variance or covariance and contrasts. See Table S3 for details.

b Biosolids × lime interaction from analysis of variance (ANOVA), slight decrease in pH and increase in EC and dead trees with BS × lime versus lime based on least square means.

c Also includes 1% lime and BS.

d Also includes 1% lime; BS; and 0%, 1%, 2.5%, or 5% BC.

e Significant BC × LSM interaction, greater increase in pH with BC and LSM.

f Very slight decrease in dead trees with 2% BS (five dead trees) versus tailings alone (six dead trees), although statistically significant, likely not meaningful.

g For survival, *p* = 0.095.

**TABLE 3:** Summary of statistical analysis for effects of lime, nutrient source, nutrient level, biochar (BC), and locally sourced microbes (LSM) on key Douglas fir and characteristics and tailing chemistry for plants grown in the Formosa Mine tailings in Experiment 2.^a^

Parameter		Main effects					Major interactions/comments
	Lime (1% vs. 0%)	Lime (1% vs. 0.5%)^b^	Nutrient source (BS vs. F)^c^	Nutrient level (2%, 0.5%, 0.25%; BS and/or F)^d^	BC (2.5% vs. 0%)^e^	LSM (yes vs. no)^f^	
Leachate pH	+	+	±	−	+	−	Nutrient source x level interaction, BS lower pH at 0.5%, and BS higher pH at 2% nutrient level.
Leachate EC	−	ns	−	+	ns^g^	ns	Biochar increased EC only at lowest nutrient level. LSM decreased EC only at 2% BS level.
Survival	ns^h^	−	−	−	+	ns	Source effect marginal. Nutrient level effect especially at 2%. Nutrient effect uncertain with LSM treatment.
New needle DW	N/A^i^	ns	ns	ns^j^	ns	ns	Biochar decreased new needle DW across nutrient sources and levels, but no effect across Lime and BS levels, thus not considered to be significant. Biochar × lime interaction, but no significant contrasts.
New Needle Ca	N/A^i^	ns	ns	+	−	ns	
New Needle Cu	N/A^i^	−	ns^g^	ns	−	ns	Nutrient source and source × level interaction significant, but meaning uncertain.
New Needle K	N/A^i^	ns	ns	ns^k^	+	ns	Many interactions. Higher lime decreased K, but only without BC, thus ns for lime. Biochar increased K at 2 lower nutrient levels, and was generally significant. BS had lower K than F, but only without BC. F had higher K than BS, only at highest nutrient level.

Abbreviations: BC, biochar; BS, biosolids; DW, dry weight; F, mineral fertilizer; LSM, Locally Sourced Microbes.

a Clear main effect by itself significant generally for at least two of the three analyses for nutrient levels and for both analyses for BC. A “+” indicates a significant increase in the parameter from the first parenthetical variable to the second, “−” significant decrease, and “ns” no effect at *p* > 0.05 according to analysis of variance and contrasts. There may be significant interactions between these parameters as indicated in comments. See Tables S4 and S5 for *p* values for main effects and interactions.

b Included different levels of BS and BC.

c Included 1% lime and different levels of BS or F, and BC.

d Included different levels of lime, BS or F, BC, and LSM.

e Included different levels of lime, BS or F, and BC.

f Included 1% lime and different levels of BS and 2.5% BC.

g Even though it was significant, the biochar effect is uncertain due to interaction with nutrient level, no significant contrast between sources, and high variability in F response.

h There was no significant difference in survival between the tailings alone and 1% lime alone treatments because of the very large standard error for the difference between treatments; that is, all dead trees for the tailings alone and 50% dead trees for the 1% lime treatment.

i Not run as no new needle DW for tailings alone.

j Mixed results, highest BS level increased new needle DW versus lowest level across lime and BC levels, but highest new needle DW with middle versus lowest and highest BS or F level across BC levels.

k Nutrient level significant for one of the three analyses and barely significant for a second analysis, with a limited increase with 2% nutrients.

## Core Ideas

- Amendments influenced Formosa Mine tailings chemistry and affected survival of Douglas fir in greenhouse studies.
- Lime decreased tailings leachate heavy metal availability, and increased pH and plant survival.
- Biosolids (BS) or fertilizer (F) provided plant nutrients, but 2% by weight resulted in phytotoxicity.
- Biochar (BC) enhanced plant survival likely by further increasing tailings pH.
- Amending with 1% lime, 0.25% or 0.5% BS or F, and 2.5% BC is recommended to establish plants on mine tailings.

## Abbreviations:

BC: biochar
BS: biosolids
EC: electrical conductivity
F: mineral fertilizer
LSM: locally sourced microbes
RO: reverse osmosis
