# Supplementary material for: Designing amendments to improve plant performance for mine tailings revegetation
Source: Agrosyst Geosci Environ. Author manuscript; Available in PMC 2024 Sep 1. (PMC10805240; doi:10.1002/agg2.20409)
Supplement: Supplement1 [file NIHMS1950329-supplement-Supplement1.pdf]

SUPPLEMENTAL MATERIAL

Designing Amendments to Improve Plant Performance for Mine Tailings Revegetation

Mark G. Johnson[a*], David M. Olszyk[a], Tamotsu Shiroyama[b] , Michael A. Bollman[a], Maliha S. Nash[c], Viola A. Manning[d], Kristin M. Trippe[d], Donald W. Watts[e], and Jeffrey M/ Novak[e]

Affiliations

[a]Pacific Ecological Systems Division, Center for Public Health and Environmental Assessment, U.S. Environmental Protection Agency, Corvallis, OR USA

[b]National Asian Pacific Center on Aging, Senior Environmental Employment Program, Corvallis, OR, USA

[c]Pacific Ecological Systems Division, Center for Public Health and Environmental Assessment, U.S. Environmental Protection Agency, Newport, OR USA

[d] National Forage Seed Production Research Center, USDA Agricultural Research Service, Corvallis, OR, USA

[e]Coastal Plain Soil, Water and Plant Conservation Research, USDA Agricultural Research Service, Florence, SC, USA

SUPPLEMENTAL TABLES

SUPPLEMENTAL TABLE S1 Key cultural dates and average daily environmental conditions (averages across all hours in growth period) for experiments to determine effects of amendments on plants grown in Formosa mine tailings.[a]

Experiment	Planted	Harvested	Max. T. °C	Min. T. °C	Avg. T. °C	PAR (μmol/m²/s)	RH (%)	CO_2 (ppm)
1 - Douglas-fir	5-May-17	23-Aug-17[b]	27.2	13.1	19.8	193	68	417
2 - Douglas-fir	20-Oct-17	12-Mar-18	20.2	10.5	15.8	152	53	433

[a]Min., minimum; Max., maximum; Avg., average; T, temperature; RH, relative humidity. Experiments begin and end at noon. Max. and min. are averages of daily average max. and min. [b]A few Douglas-fir were harvested 24-Aug-17.

SUPPLEMENTAL TABLE S2 Number of replicates per treatment for new needle dry weight and elements for live trees in Experiment 2 out of 10 possible replicates. Includes 0 values. Abbreviations: T, tailings; L, lime; BS, biosolids; F, chemical fertilizer; BC, biochar; LSM, Locally Sourced Microbes.

Treatment	Dry Weight	Elements		Treatment	Dry Weight	Elements
T	0	0		T+0.5%L+0.5%BS+2.5%BC	10	10
T+1%L	5	3		T+0.5%L+2%BS+2.5%BC	8	8
T+1%L+0.25%BS+0%BC	5	5		T+1%L+0.25%F+0%BC	7	7
T+1%:+0.5%BS+0%BC	3	3		T+1%L+0.5%F+0%BC	8	8
T+1%:+2%BS+0%BC	1	1		T+1%L+2%F+0%BC	1	0
T+1%:+0.25%BS+2.5%BC	10	8		T+1%L+0.25%F+2.5%BC	8	8
T+1%:+0.5%BS+2.5%BC	7	7		T+1%L+0.5%F+2.5%BC	10	9
T+1%:+2%BS+2.5%BC	4	4		T+1%L+2%F+2.5%BC	5	4
T+0.5%L+0.25%BS+0%BC	7	7		T+1%L+0%BS+2.5%BC+LSM	7	5
T+0.5%L+0.5%BS+0%BC	8	8		T+1%:+0.25%BS+2.5%BC+LSM	6	6
T+0.5%L+2%BS+0%BC	2	2		T+1%:+0.5%BS+2.5%BC+LSM	6	6
T+0.5%L+0.25%BS+2.5%BC	8	7		T+1%:+2%BS+2.5%BC+LSM	5	5

SUPPLEMENTAL TABLE S3 The p values from Analysis of Covariance and Variance for tailing pH, EC and dead trees (as measure of survival) for Douglas fir plants grown in Formosa mine tailings in Experiment 1.[a] Parameter statistics were based on Analysis of Covariance and Variance for all parameters except tree survival (measured as number of dead trees), where analysis was by logistic regression and Chi-squared tests.

Comparing control, lime and biosolids treatments.

	pH	EC	Survival
Intercept	<0.001	0.972	N/A
Biosolids	<0.001	0.000	0.023
Lime	<0.001	0.184	0.002
Block	0.550	0.723	0.384
Preheight	Dropped	0.024	N/A
Biosolids x Lime	<0.001	0.019	0.001
Lime x Preheight	Dropped	0.092	N/A

Comparing biochar and LSM treatments.

	pH	EC	Survival
Intercept	<0.001	<0.001	N/A
Biochar	<0.001	<0.001	0.095
LSM	<0.001	0.931	0.189
Block	0.296	0.888	0.295
Preheight	Dropped	Dropped	N/A
Biochar x LSM	<0.001	Dropped	N/A

[a]Values are Pr(>F) except for tree survival Pr(>Chisq). Abbreviations: EC, electrical conductivity; LSM, Locally Sourced Microbes. The N/A (not applicable) indicates that factor was not included in the final analysis. Dropped refers to factors that were not included in the final model. Intercept tests if the overall mean response differs from zero.

SUPPLEMENTAL TABLE S4 The p values from Analysis of Covariance and Variance for tailing pH and EC and plant responses for Douglas-fir plants grown in Formosa mine tailings in Experiment 2. Parameter statistics were based on Analysis of Covariance and Variance for all parameters except tree survival (measured as number of dead trees), where analysis was by logistic regression and Chi-squared tests.[a]

Comparing lime, biochar, and nutrient level for biosolids without LSM.

	pH	EC	Survival	New Needle DW[b]
Intercept	0.000	0.000	N/A	0.596
Biochar	0.000	0.000	0.000	0.392
Lime	0.000	0.069	0.002	0.109
Nutrient Level	0.000	0.000	0.000	0.014
Block	0.647	0.262	0.048	0.608
Biochar x Lime	Dropped	Dropped	N/A	0.033
Biochar x Nutrient Level	Dropped	0.000	N/A	Dropped
Lime x Nutrient Level	Dropped	0.005	N/A	Dropped
Preheight	Dropped	Dropped	N/A	Dropped

Comparing biosolids vs. fertilizer, nutrient level, and biochar at 1% lime without LSM.

	pH	EC	Survival	New Needle DW[b]
Intercept	0.000	0.000	N/A	0.001
Biochar	0.000	0.001	0.000	0.002
Nutrient Level	0.000	0.000	0.000	0.001

Nutrient Source	0.017	0.008	0.031	0.212
Block	0.053	0.367	0.060	0.150
Biochar x Nutrient Level	Dropped	0.004	N/A	Dropped
Nutrient Source x Level	0.000	Dropped	N/A	Dropped
Preheight	Dropped	Dropped	N/A	0.0000

Comparing LSM and nutrient level at 1% lime, biosolids and 2.5% biochar

	pH	EC	Survival	New Needle DW[b]
Intercept	0.000	0.000	N/A	0.135
LSM	0.020	0.893	0.217	0.282
Nutrient Level	0.000	0.000	0.038[c]	0.189
Block	0.485	0.006	0.242	0.814
LSM x Nutrient Level	Dropped	0.019	N/A	Dropped
Preheight	Dropped	Dropped	N/A	0.112

[a]Values are Pr(>F) except for tree survival Pr(>Chisq). Abbreviations: EC, electrical conductivity; NE, needle; DW, dry weight; RT, root; LSM, Locally-Sourced Microbes. The N/A (not applicable) indicates that factor not included in the final analysis. Dropped refers to factors that were not included in the final model. Intercept tests if the overall mean response differs from zero. For dead trees is Pr(>Chisq).

[b]New needles weights only for live trees if no weight used 0.

[c]Even though nutrient level slightly significant, results uncertain due to difficulty in determining contracts between nutrient levels.

SUPPLEMENTAL TABLE S5 The p values from Analysis of Covariance and Variance for tailing leachate and new needle elemental responses for Douglas-fir plants grown in Formosa mine tailings in Experiment 2. Note only trees with needle growth included.[a]

Comparing lime, biochar, and nutrient level for biosolids without LSM.

	Leachate K	Leachate Zn[b]	New NE Ca	New NE Cu	New NE K	New NE Mg	New NE P
Intercept	0.000	0.119	0.000	0.004	0.000	0.000	0.000
Biochar	0.000	0.000	0.002	0.035	0.000	0.026	0.267
Lime	0.189	N/A	0.160	0.000	0.000	0.952	0.660
Nutrient Level	0.000	0.436	0.000	0.343	0.000	0.001	0.000
Block	0.796	0.584	0.078	0.587	0.343	0.067	0.232
Biochar x Lime	0.464	N/A	Dropped	Dropped	0.020	Dropped	Dropped
Biochar x Nutrient Level	0.000	0.003	Dropped	Dropped	0.002	Dropped	Dropped
Lime x Nutrient Level	0.669	N/A	Dropped	Dropped	Dropped	Dropped	Dropped
Biochar x Lime x Nutrient Level	0.000	N/A	Dropped	Dropped	Dropped	Dropped	Dropped
Preheight	Dropped	N/A	Dropped	Dropped	0.067	Dropped	Dropped

Comparing biosolids vs. fertilizer, nutrient level, and biochar at 1% lime without LSM.

	Leachate K	Leachate Zn	New NE Ca	New NE Cu	New NE K	New NE Mg	New NE P
Intercept	0.046	N/A	0.000	0.058	0.000	0.000	0.000
Biochar	0.000	N/A	0.000	0.031	0.000	0.000	0.395
Nutrient Level	0.000	N/A	0.000	0.884	0.262	0.120	0.245
Nutrient Source	0.022	N/A	0.664	0.017	0.847	0.004	0.013
Block	0.874	N/A	0.519	0.343	0.645	0.183	0.209

Biochar x Nutrient Level	0.001	N/A	Dropped	Dropped	0.008	Dropped	Dropped
Biochar x Nutrient Source	0.035	N/A	Dropped	Dropped	0.039	Dropped	Dropped
Nutrient Source x Level	0.016	N/A	Dropped	0.021	0.000	Dropped	0.000
Preheight	Dropped	N/A	Dropped	0.037	Dropped	Dropped	Dropped

Comparing LSM and nutrient level at 1% lime, biosolids and 2.5% biochar

	Leachate K	Leachate Zn	New NE Ca	New NE Cu	New NE K	New NE Mg	New NE P
Intercept	0.000	N/A	0.000	0.238	0.000	0.000	0.000
LSM	0.891	N/A	0.109	0.979	0.325	0.550	0.522
Nutrient Level	0.000	N/A	0.000	0.171	0.034	0.262	0.009
Block	0.596	N/A	0.569	0.669	0.698	0.271	0.312
LSM x Nutrient Level	Dropped	N/A	Dropped	Dropped	Dropped	Dropped	Dropped
Preheight	Dropped	N/A	Dropped	Dropped	Dropped	Dropped	Dropped

[a]Values are Pr(>F). Abbreviations: EC, electrical conductivity; NE, needle; K, potassium; Zn, zinc; Ca, calcium; Cu, copper; Mg, magnesium; Locally Sourced Microbes. The N/A (not applicable) indicates that factor not included in the analysis as analysis was limited. Dropped refers to factors that were not included in the final model. Intercept tests if the overall mean response differs from zero.

[b]Limited model considering only biochar and nutrient level for biosolids and 0.5% lime.

SUPPLEMENTAL FIGURES

SUPPLEMENTAL FIGURE S1 Boxplots of effects of amendment treatments on Formosa mine tailings pH (a) and EC (b) for Douglas fir seedlings in Experiment 1. T, tailings; L, lime; BS, biosolids; BC, Biochar; LSM, Locally Sourced Microbes. N=6. Based on regression analysis: pH (no LSM) = 6.793 + 1.853 * BC^1 – 0.684 BC^2; and pH (LSM) = 6.892 – 0.950 * biochar^1 - 0.387 BC^2 (variance 0.009 around both lines). Across LSM treatments EC = 2.773 – 2.691 * BC^1 + 0.657 * BC^2 + 0.505 * BC^3 (variance 0.108). Addition of BC to the lime and BS increased pH with a quadratic regression response to BC (a), and a decrease in EC with a cubic regression response to BC (b). The LSM treatment slightly increased the BC effect on pH as shown by the larger x-intercepts for the regression equations (a).

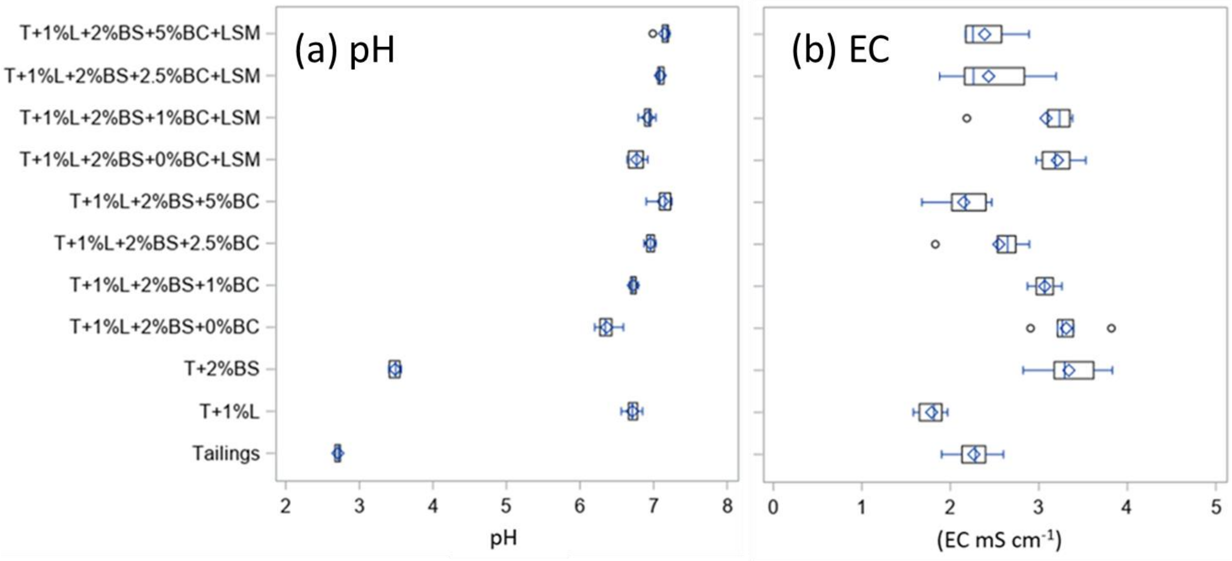

SUPPLEMENTAL FIGURE S2 Boxplot for effects of amendments on new growth dry weight (needles plus stems) for Douglas fir plants grown in Formosa mine tailings in Experiment 1. Data only for live trees, except that trees alive but without new growth not included. Abbreviations: T, tailings; L, lime; BS, biosolids; BC, Biochar; LSM, Locally Sourced Microbes. No statistics performed. N=6 for T+1% Lime, N=5 for T+1%L+2%BS+5%BC+LSM, N=4 for T+1%L+2%BS+2.5%BC, N=3 for T+1%L+2%BS+5%BC, T+1%L+2%BS+0%BC+LSM, and T+1%L+2%BS+2.5%BC+LSM; N=2 for T+1%L+2%BS+1%BC, N=1 for T+2%BS, T+1%L+2%BS+0%BC and T+1%L+2%BS+1%BC+LSM; and N=0 for Tailings alone.

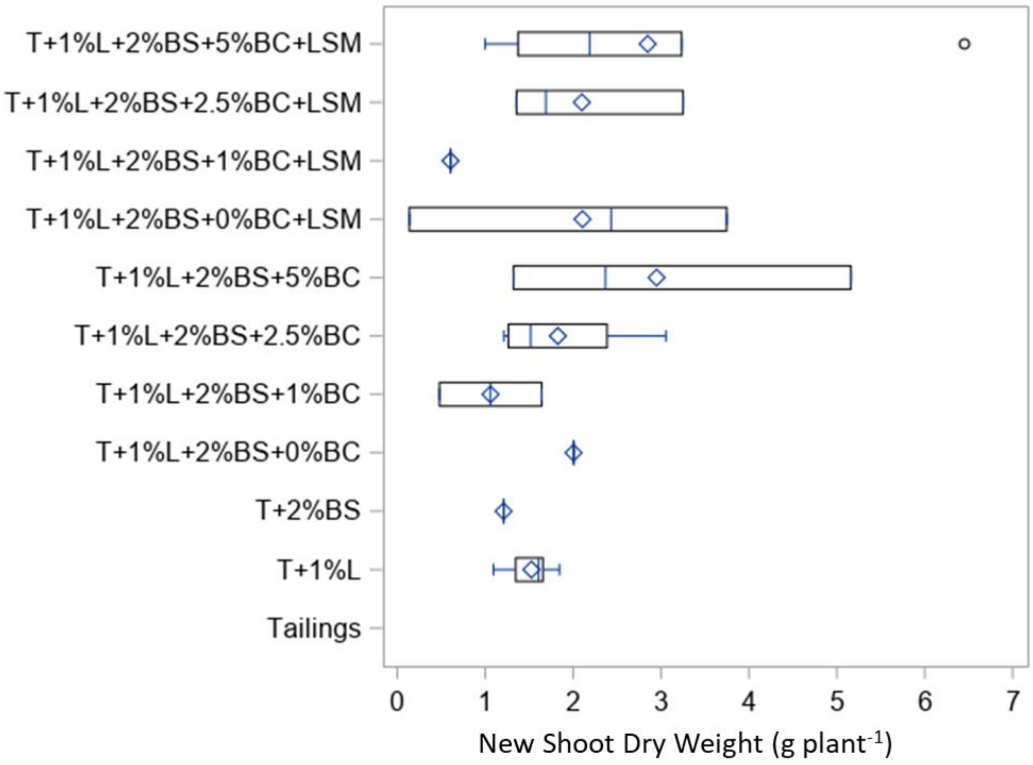

SUPPLEMENTAL FIGURE S3 Boxplots for effects of amendments on new needle Ca for Douglas fir plants grown in Formosa mine tailings in Experiment 2. Abbreviations: BS=biosolids, F=fertilizer, LSM=Locally Sourced Microbes. Black horizonal line in bar is median, cross hatch in bar is 2.5% BC, the darker the color the higher the BS or F level. For N, see Supplemental Table S2.

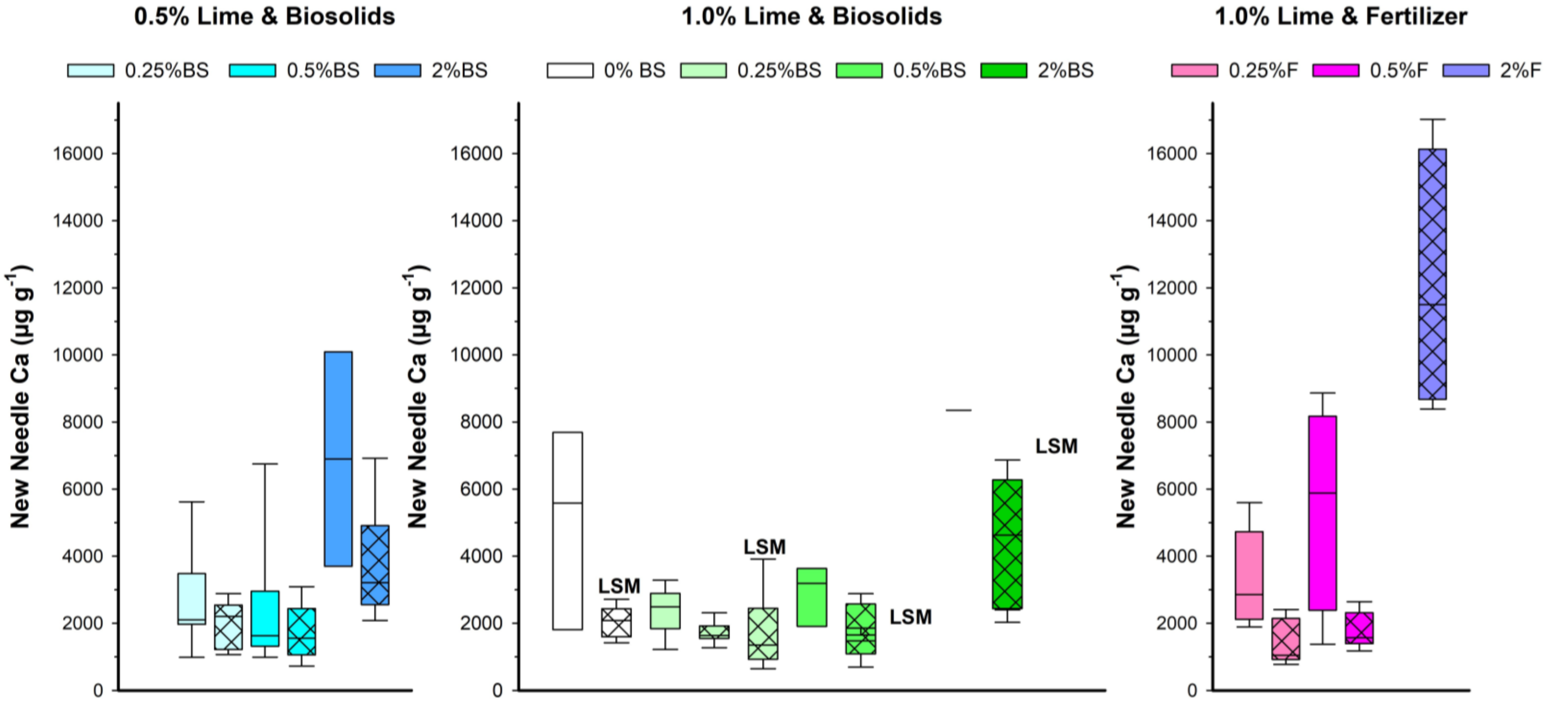

SUPPLEMENTAL FIGURE S4 Boxplots for effects of amendments on new needle Cu for Douglas fir plants grown in Formosa mine tailings in Experiment 2. Abbreviations: BS=biosolids, F=fertilizer, LSM=Locally Sourced Microbes. Black horizontal line in bar is median, cross hatch in bar is 2.5% BC, the darker the color the higher the BS or F level. For N, see Supplemental Table S2.

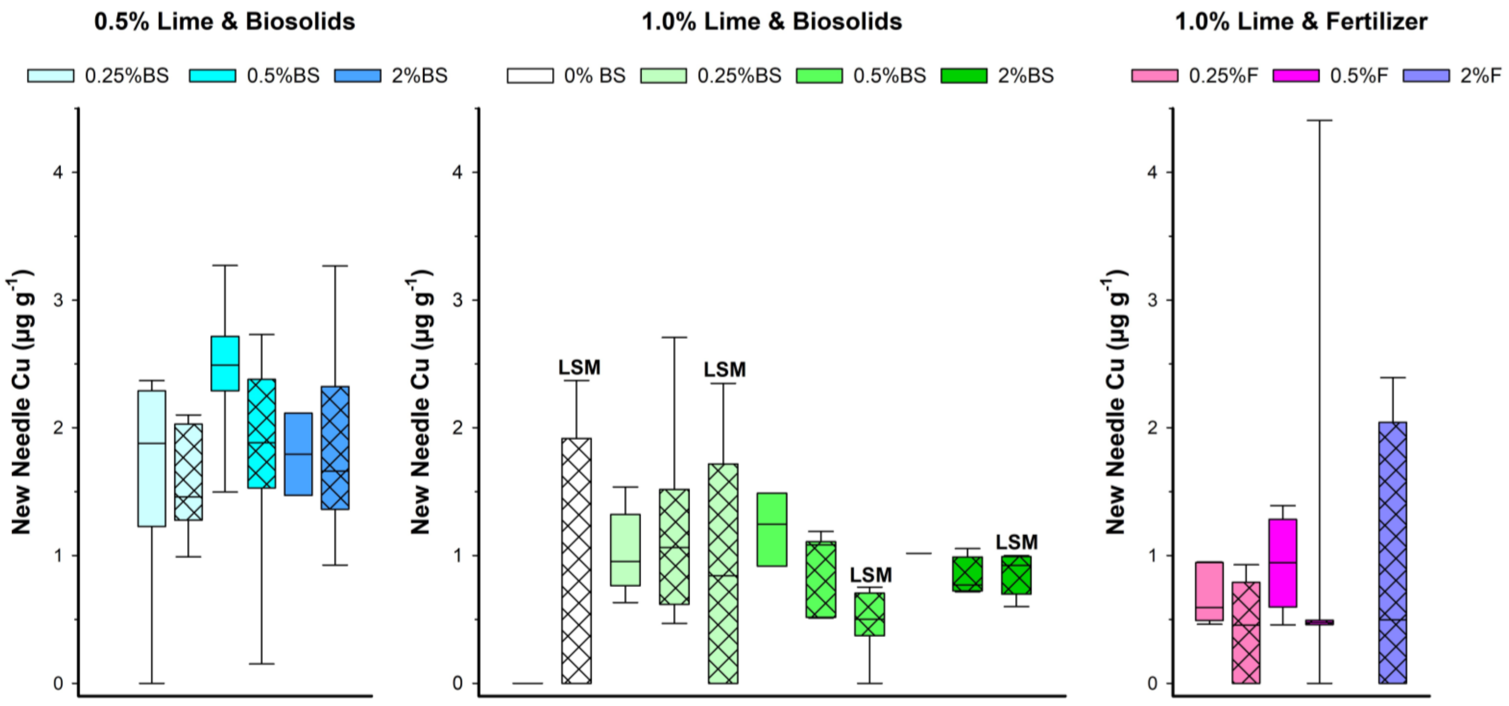

SUPPLEMENTAL FIGURE S5 Boxplots for effects of amendments on leachate electrical conductivity (EC) for Douglas fir plants grown in Formosa mine tailings in Experiment 2. Abbreviations: BS=biosolids, F=fertilizer, LSM=Locally Sourced Microbes. Black horizontal line in bar is median, cross hatch in bar is 2.5% BC, the darker the color the higher the BS or F level, and orange bar in the far left of 0.5% lime and BS panel is the tailings without amendments. N=10.

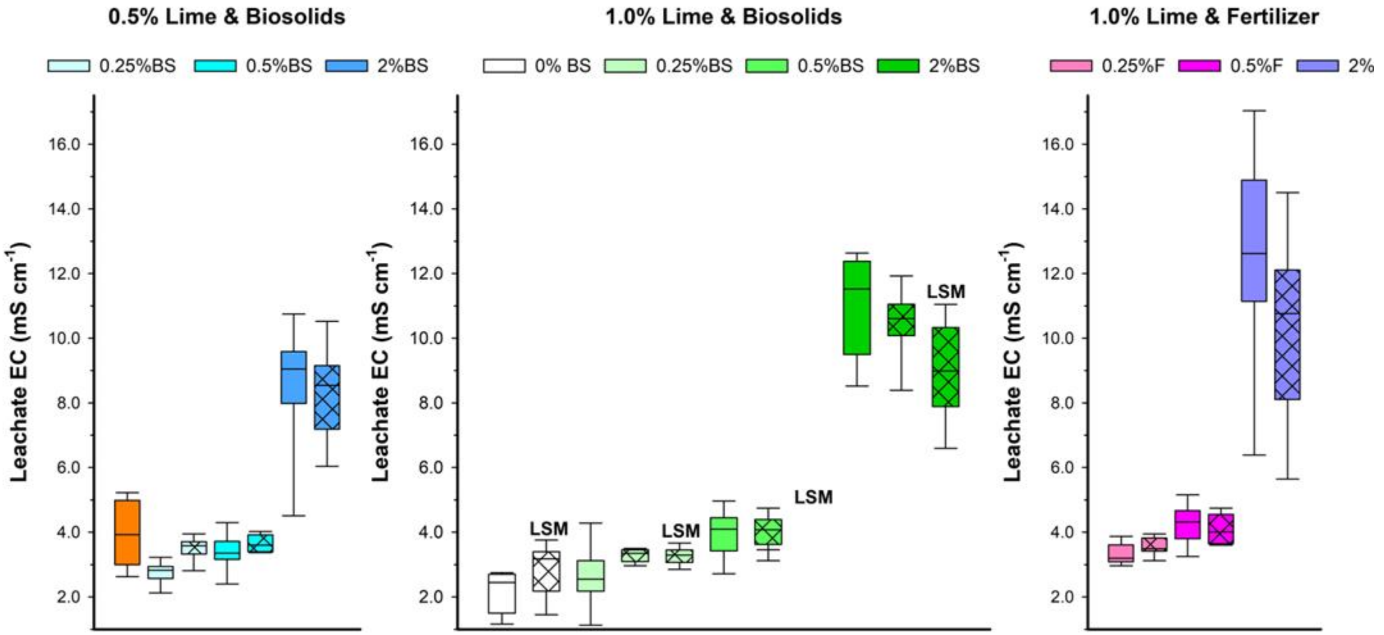

SUPPLEMENTAL FIGURE S6 Boxplots for effects of amendments on leachate Zn for Douglas fir plants grown in Formosa mine tailings in Experiment 2. Abbreviations: BS=biosolids, F=fertilizer, LSM=Locally Sourced Microbes. Black horizontal line in bar is median, cross hatch in bar is 2.5% BC, the darker the color the higher the BS or F level, and orange bar in the far left of 0.5% lime and BS panel is the tailings without amendments. N=10.

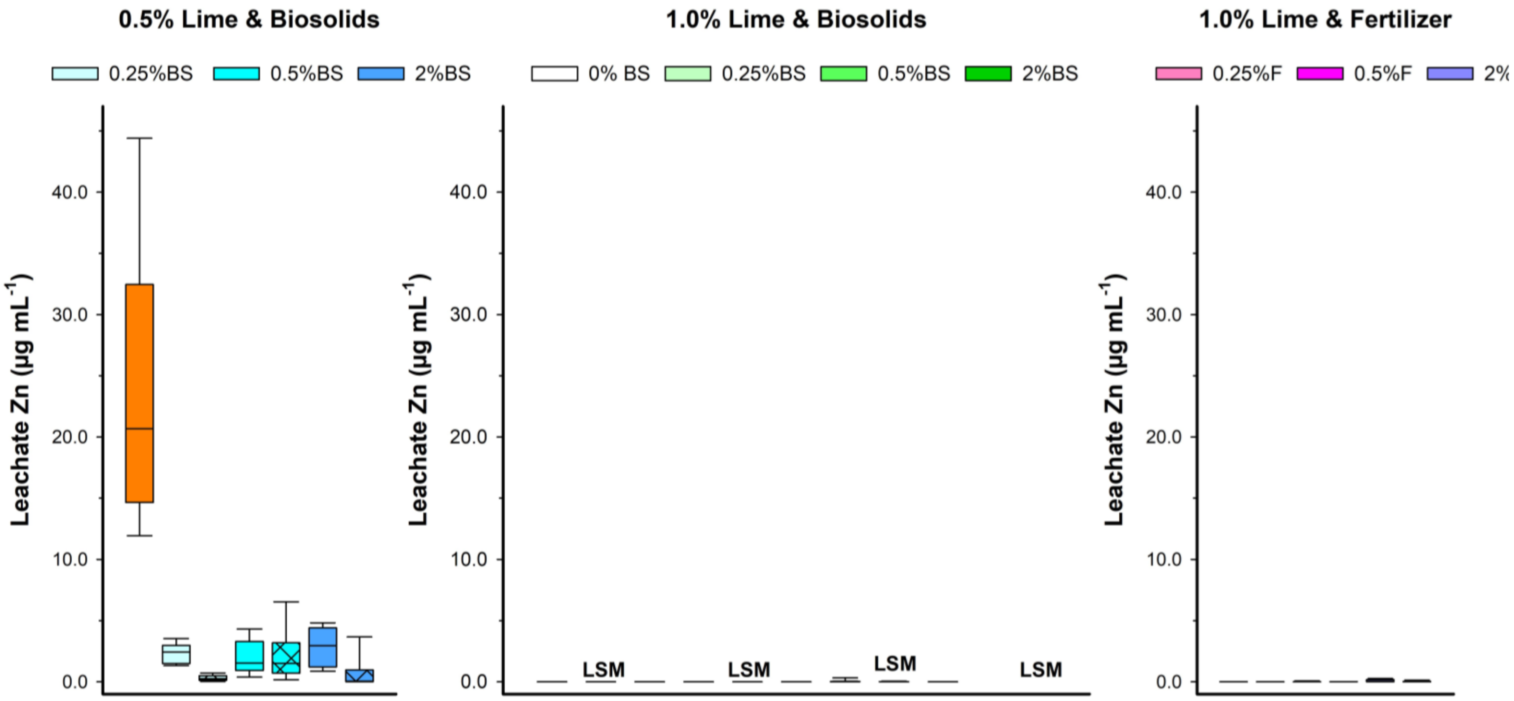

SUPPLEMENTAL FIGURE S7 Boxplots for effects of amendments on leachate K for Douglas fir plants grown in Formosa mine tailings in Experiment 2. Abbreviations: BS=biosolids, F=fertilizer, LSM=Locally Sourced Microbes. Black horizontal line in bar is median, cross hatch in bar is 2.5% BC, the darker the color the higher the nutrient level, and flat bar in far lower left of 0.5% lime and BS panel is the tailings without amendments. N=10.

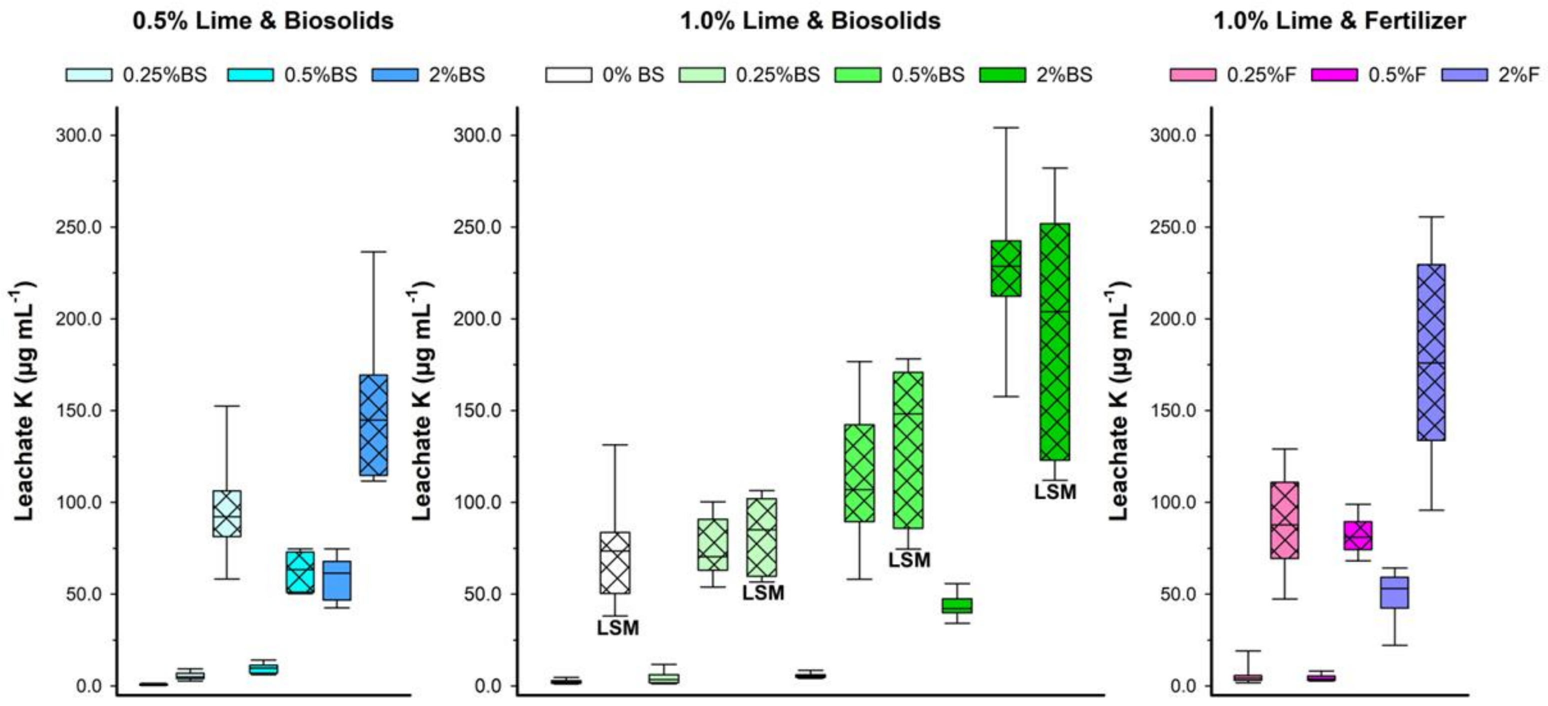

SUPPLEMENTAL TEXT S1

Preliminary Tailing and Lime Study

Background for selecting 1% lime as the initial pH regulating treatment for Formosa field studies

The pH of unamended Formosa mine tailings was approximately 3. The pH of soil from the reference site (target) was approximately 5.5. Calculations indicated that about 2.8% lime would be necessary to ameliorate the total reserve acidity in the tailings (J. Ippolito, personal communication), and preliminary experiments had suggested that short-term pH adjustment to about pH 7 could be obtained with the addition of about 0.8% lime (J. Novak, personal communication). To determine an optimum lime level to increase the tailings pH to that of the reference site, we conducted a 71-day liming test (Figure 1) using a range of lime additions from 0.56% to 3.08% (fractions of 0.2 to 1.1 of 2.8%). For this length of time, there were no discernable differences on pH among lime treatments greater than 0.84%. Lime addition of 0.56% slowly dropped in pH over the course of the test.

To estimate the potential effects that the other tailing amendments (biosolids and biochar) might have on pH, both alone and with other amendments, we conducted a shorter (month-long) full factorial test using 2% biosolids, 2.5% biochar, and 0.5% lime (Figure 2). Results indicated that biosolids and biochar together brought pH up to about 4 initially and slowly increased over 4 weeks to nearly 5. Biosolids plus biochar plus 0.5% lime brought the tailing pH up to about 7. Similar to the previous test, lime-only addition of 0.5% dropped in pH over the course of the test.

Finally, we conducted a third test for a longer time period (~160 days) using 2% biosolids + 2.5% biochar and a narrower range of lime additions (Figure 3). For this length of time, there

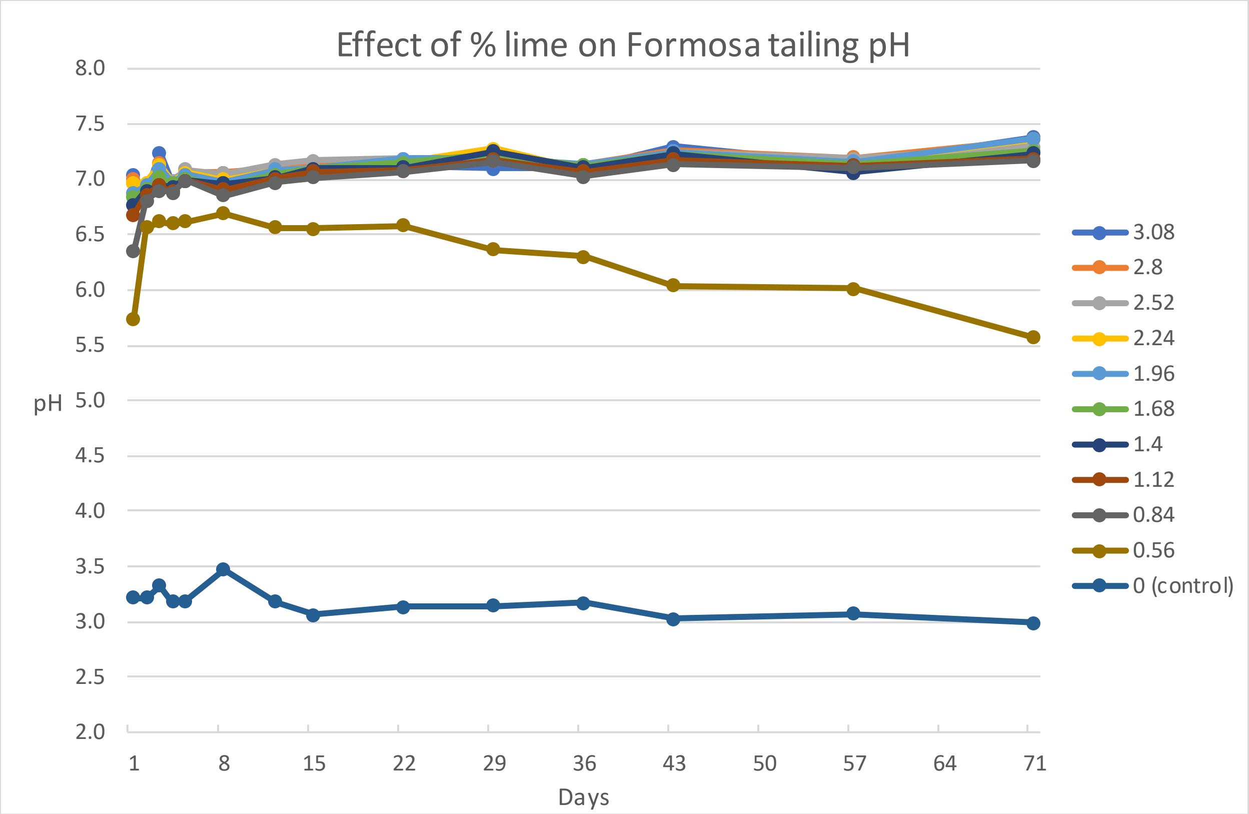

FIGURE 1 Results from 71 day test on effects of different lime concentrations on tailing pH.

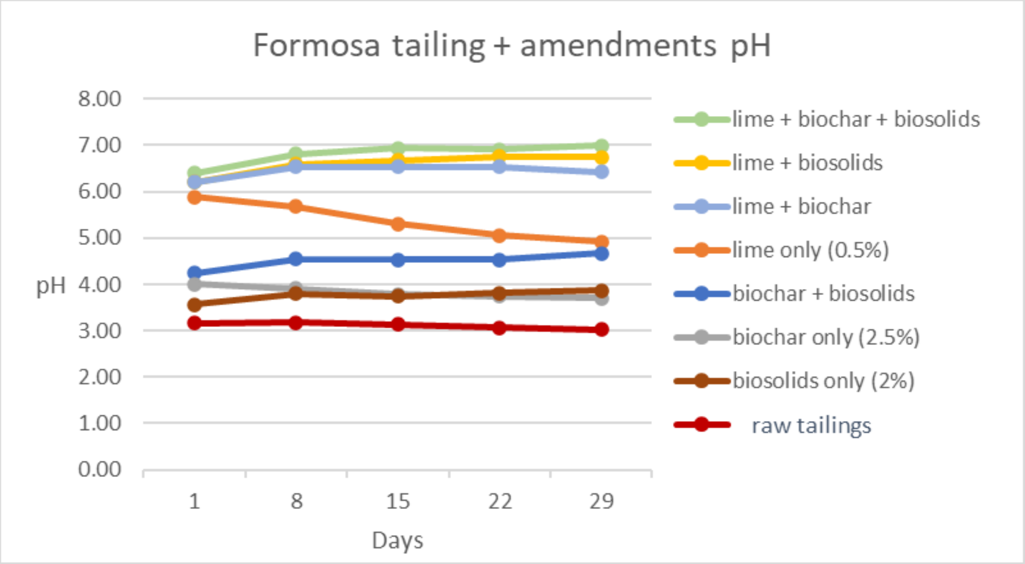

FIGURE 2 Effects of combinations of amendments on tailing pH.

were no discernable differences in pH among lime treatments greater than or equal to 0.8% and pH remained relatively stable at about 7.4. The activity of the biosolids and biochar without lime stabilized at about pH 5 after 5 weeks.

Based on these tests, and considering that the pH achieved with lime additions over 0.8% would be well above the reference soil target, and also considering that some pH amelioration would come from the biosolids and biochar, we decided to recommend 1% lime to remediate the low pH of Formosa mine tailings, with an additional recommendation to monitor the pH over time and add additional lime should the pH drop close to the reference soil target.

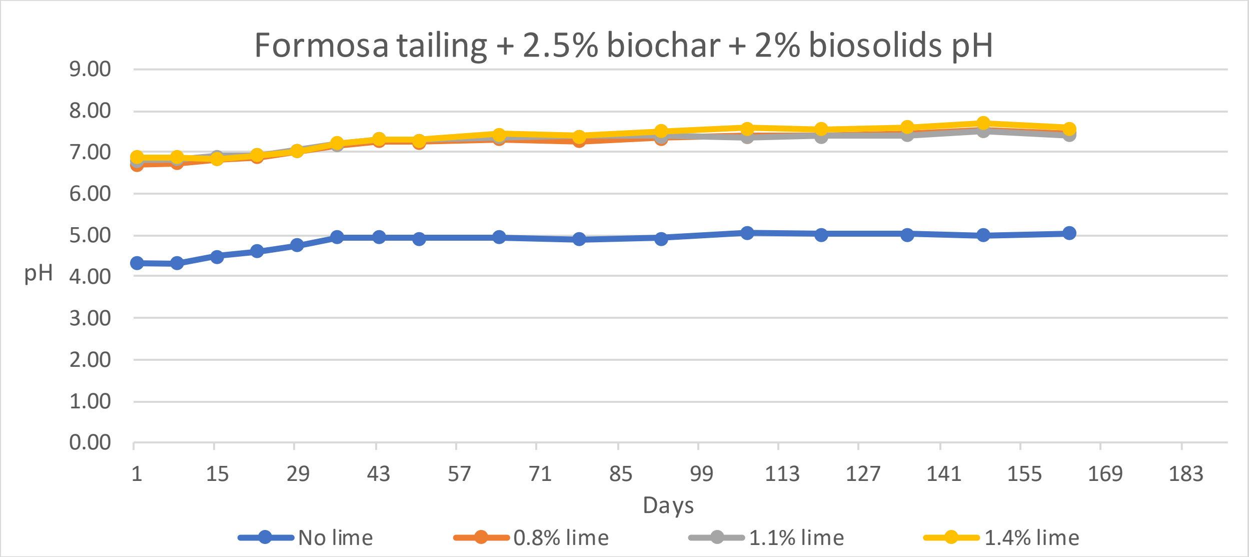

FIGURE 3 Effects of a narrow range of lime concentrations on tailing pH.

SUPPLEMENTAL TEXT S2

Details for Statistical Analysis Procedures

Experiment 1 was an initial exploratory study to determine if a specific set of amendments enhanced plant growth in the Formosa mine tailings. Treatments used in Experiment 1 are shown in Supplemental Figure S1a. There were six replicate containers per treatment in blocks, with replicate containers for all Douglas fir and blue wildrye treatments randomly located across all plastic holders on the greenhouse bench. Boxplots were used to indicate the range of values observed and were also used to identify potential outliers. The experiment was designed to address five questions: (1) Does 1% lime alone affect plants? (2) Does 2% biosolids alone affect plants? (3) Does the addition of biosolids affect the response to lime? (4) Does the addition of biochar affect the response to biosolids and lime? (5) For Douglas fir, do Locally Sourced Microbes (LSM) affect the response to the mixture of lime, biosolids and biochar?

Experiment 2 was designed to determine how different levels of lime and biosolids, type of nutrient source, and biochar and LSM affect Douglas fir seedling growth in order to better predict the response in the field. Experiment 2 was a randomized complete block design where Douglas fir seedlings were randomly assigned one of twenty-four treatments within each of ten blocks. The blocks were arranged in two groups of five on a greenhouse bench. The twenty-four treatments are shown in Supplemental Table S2. The statistical analysis addressed four questions of interest for each response parameter: (1) Is what is the effect of 1% lime alone *vs.* the unamended tailings on a response parameter? (2) Is there a difference in the effect of 0.5% or 1% lime on a response parameter with different levels of biosolids without biochar or with biochar? (3) Is there a difference in the effect of biochar on the response parameter for the different sources of nitrogen (fertilizer or biosolids)? Is there a difference in the effect of

nitrogen on the response parameter for the different sources of nitrogen when biochar is not present? If so, by how much do they differ? (4) Is there a difference in the effect of biosolids on the response parameter when LSM is present or absent, when 1% lime and 2.5% biochar are present? The 1% lime + 0% BS + BC + LSM was an extra treatment which was not used for analyses.

For Experiment 2, to compare the tailings alone and tailings + 1% lime treatments a linear mixed model for the Analysis of Variance (ANOVA) was used, with a Tukey adjusted and adjusted p value to determine if there was a significant difference between means to account for any unbalanced number of measurements per treatment. Data transformations were based on those used for the ANCOVA analysis described later, but a covariate was not used. A logistic regression was used for survival.

The data for multiple treatments in each experiment were explored prior to each Analysis of Covariance (ANCOVA) or Analysis of Variance (ANOVA) to investigate if any transformations were necessary to allow the data to meet the validity conditions of an ANCOVA or ANOVA, using statistical tests and/or appropriate diagnostic plots such as boxplots. The randomized complete block design of the project satisfied the validity condition of independence of error terms. The homogeneity of variances validity condition was investigated using boxplots, residuals vs fitted values plot, scale-location plot, and a score test; ncvTest() in the car R package performs a score test for non-constant error variance (Fox and Weisberg, 2019).

The normality of errors validity condition was checked using a QQ plot and/or a histogram of residuals as well as a Shapiro-Wilk Test for normality. Deviations from normality may have been deemed minor due to the balanced nature of the design and the total sample size of the experiment. If a problem was detected, a transformation T_1 (see Equation 1) among the scaled

power family (Box-Cox power family) of transformations was estimated using the powerTransform() function in the car R package (Fox and Weisberg, 2019).

(Equation 1) $$\boldsymbol{T_1(x,\lambda)} = \begin{cases} \frac{x^{\lambda}-1}{\lambda}, \ \lambda \neq 0 \\ \ln(x), \ \lambda = 0 \end{cases}$$

If negative values or zero values were present, then the Box-Cox family of transformations proposed by Hawkins and Weisberg (Fox and Weisberg, 2019) was used (see Equation 2).

(Equation 2) $$\boldsymbol{T_2(x,\lambda,\gamma)} = \begin{cases} \frac{\left[\frac{1}{2}\left(x+\sqrt{x^2+\gamma^2}\right)\right]^{\lambda}-1}{\lambda}, \ \lambda \neq 0 \\ \ln\left(\frac{1}{2}\left(x+\sqrt{x^2+\gamma^2}\right)\right), \ \lambda = 0 \end{cases}$$

To paraphrase from Fox and Weisberg (2019), regression models are approximations and the transformed response can help to make for a better approximation. Final judgement on transformations were made by the statistician based on how well the data or transformed data met the validity conditions.

Least squared means (LS means; also referred to as estimated marginal means) and standard errors were calculated from each ANCOVA or ANOVA model fit. The LS means were calculated on the transformed scale and then back-transformed. For the ANCOVA models, which had pre-height included as a predictor, the LS means were calculated at the average pre-height observed.

Back-transformations are calculated using inverse functions. The inverse transformation of Equation 1 is

(Equation 3) $$\boldsymbol{T_1^{-1}(z,\lambda)} = \begin{cases} (1+\lambda \cdot z)^{1/\lambda}, \ \lambda \neq 0 \\ \exp(z), \ \lambda = 0. \end{cases}$$

The (-1) attached to the name of the transformation (T_1^{-1}) is meant to emphasize that the transformation is an inverse transformation. The expression exp(z) refers to raising the real number e (≈2.718) to the power of z (i.e. e^z). The inverse transformation of Equation 2 is

(Equation 4) $$\boldsymbol{T_2^{-1}(z, \lambda, \gamma)} = \begin{cases} \frac{(2\cdot(1+\lambda\cdot z)^{1/\lambda})^2-\gamma^2}{4\cdot(1+\lambda\cdot z)^{1/\lambda}}, & \lambda \neq 0 \\ \frac{(2\cdot\exp(z))^2-\gamma^2}{4\cdot\exp(z)}, & \lambda = 0. \end{cases}$$

γ in Equation 4 is a small positive number. The general formulas for the back-transformations were computed by hand here, however for a given λ and γ the specific equation can be obtained using the make.tran() function in the emmeans R package (Lenth, 2020).

The means obtained from back-transforming LS means are generalized means. These generalized means are acceptable for presenting when the goal is simply to make inferences comparing treatments (i.e. one mean is less than another mean). However, if the goal is to report results that are meant to reflect the expected value of the non-transformed response, then a bias adjustment should be used (Fox and Weisberg, 2019).

The generalized means obtained from back-transforming the LS means using the inverse transformations (Equation 3 and Equation 4) could result in transformation bias in the back-transformed estimates. For data that were right-skewed and transformed to be more symmetric, the back-transformed estimates could be biased low which could be misleading. Similarly, if data were left-skewed and transformed to be more symmetric, the back-transformed estimates could be biased high. The severity of the transformation bias that could have been observed is dependent on the residual standard deviation of the model. If the residual standard deviation of the model is low, then the bias from back-transforming would be small. The emmeans R package currently allows for second-order bias adjustment correction if a bias-adjustment is requested.

The second-order bias adjustment correction is approximately (1/2·h^"? (η)·σ^2) where σ is the residual standard deviation of the model, η is derived by solving the second-order Taylor expansion for an LS mean on transformed scale, and h" is the second derivative of the inverse transformation (Fox and Weisberg, 2019). No bias adjustment corrections were performed in this analysis with the goal being to compare LS means across treatments.

When λ=-1, the back-transformed LS means may have computational concerns. Another approach can be used to make the estimates more accurate by using a generalized linear model with a Gamma family for which the canonical link function is the inverse function (i.e. $y=x^{-1}$). Unlike linear models, iterated weighted least-squares is used to obtain maximum-likelihood estimates for generalized linear models (Fox and Weisberg, 2019). Other λ values have the disadvantage of not having a family of distributions for which a canonical link function exists.

For Douglas fir (DF) the covariate, preheight, was investigated to see if there was a linear relationship with the response and if that linear relationship was the same across groups to satisfy the validity condition of homogeneous regression slopes across treatments. When preheight was removed from the model, an ANOVA model was fit and the homogeneity of regression slopes validity condition no longer applied. In some instances, there were no values due to missing or omission of questionable data, and no attempt was made to impute values.

For Experiment 1, two analysis of ANCOVA models were performed for the five questions of interest for each continuous, response parameter for DF. The sole exception being the model for height relative growth rate (HtRGR) where preheight is inherited in the response parameter.

The first three questions pertaining to this study explored the necessity for lime and/or biosolids among the nutrients fed to the seedlings. An ANCOVA model performed over the first

four treatment groups help answer the three questions to investigate the effects of lime or biosolids or both being present on the response parameter. For DF, the fullest multiple linear regression (MLR) model considered included an interaction between the predictors of the presence of lime, the presence of biosolids, and preheight; the most reduced model considered was an additive ANOVA model which excluded preheight and did not include an interaction between the lime and biosolid predictors. To account for the experimental design, all models considered accounted for block.

Model selection was performed using stepwise regression where in each step a predictor is considered for inclusion or exclusion based on a criterion established prior to model-fitting. For this project, the Bayesian information criterion (BIC), a criterion based on finding models with lower BICs, was used to help compare models and select predictors. This was done in R using the step() function in the stats R package (R core team, 2018). The BIC introduces a penalty term based on the number of observations and number of parameters which are then added to the negative log likelihood, thus it has the benefit of choosing less complex models. Lime presence and biosolids presence were not to be dropped due to the question of interest regarding their effects, and the blocking variable was retained due to the study design.

For DF, two models were performed to serve to answer the last two questions of interest which investigated the effects of biochar level and LSM presence on the response parameter when biosolids and lime are both present. The first multiple linear regression (MLR) model was fit treating biochar level as a factor. The second MLR model fit up to a cubic term, treating biochar as a continuous variable. These models were only performed for DF seedlings due to the inclusion of a LSM predictor. The data were filtered to Treatment groups 4 through 11 prior to model fitting. The first full MLR model considered included an interaction between the

predictors of biochar level, LSM presence, and preheight in the model, and the most reduced model was an additive ANOVA which excluded preheight; both models account for block.

The second full MLR model considered included an interaction between the predictors of biochar, biochar2, and biochar3 with LSM presence and preheight in the model, and the most reduced model was an MLR with LSM presence and biochar (no interaction) and which excluded preheight; both models account for block. In the first MLR model, biochar and LSM presence were not dropped due to the question of interest regarding their effects, and the blocking variable was retained due to the study design. The second MLR model allows for LSM and block to be dropped if not statistically significant at the 5% significance level; this was done to simplify discussion of the results of the regression model and because of primary interest was the effect of biochar on the response parameter.

The terms of biochar, biochar2, and biochar3 were originally input into the second MLR model as non-orthogonal terms to allow for model selection to be performed on the higher degreed terms. Model selection was performed for both models using the BIC. The second MLR model was refit using an orthogonal polynomial of the degree chosen from model selection with the poly() function in the stats R package (R core team, 2018). After the models were chosen and fit, Type III sums of squares F-tests were calculated to investigate the presence of effects among the predictors. The first MLR model was used to obtain contrasts using least squares means and a Šidák correction to counteract the potential problem with multiple comparisons. A coefficient table was provided for the second MLR model which showed the estimated effects of each level of each predictor as well as the slope, quadratic, and/or cubic effect of biochar, while accounting for all other aspects of the model.

Model selection was performed on both the ANOVA model and the MLR model using the BIC. As was done with the models for DF, the terms of biochar, biochar2, and biochar3 were first input into the MLR model as non-orthogonal terms to allow for model selection to be performed on the higher degreed terms. After model selection was performed on the MLR, the model was refit using an orthogonal polynomial of the degree chosen from model selection.

The ANOVA model chosen from model selection was used to obtain contrasts using least squares means and a Šidák correction to counteract the potential problem with multiple comparisons. A coefficient table was provided for the MLR model chosen from model selection which showed the estimated effects of each level of each predictor as well as the slope, quadratic, and/or cubic effect of biochar, while accounting for all other aspects of the model.

For Experiment 2, three ANCOVA models were performed for the respective questions of interest for each continuous response parameter. In some instances, there were no values due to missing or omission of questionable data, and no attempt was made to impute values. The first ANCOVA performed investigated the effects of lime, nutrient level, and biochar on the response parameter. The initial MLR model considered included a three-way interaction using 12 of the treatments with predictors of lime level (0.5 or 1%), nutrient level (0.25, 0.5 or 2% biosolids), and biochar level (no or 2.5% biochar) with each predictor treated as a factor, while also allowing for a possible effect for tree preheight in the model. The fertilizer and LSM treatments were not used. The most reduced model considered was an additive analysis of ANOVA model with lime level, nutrient level, and biochar level.

The second ANCOVA investigated the effects of nutrient source, nutrient level, and biochar on the response parameter. The initial MLR model considered included a three-way interaction with 12 treatments with the predictors of nutrient source (biosolids or fertilizer), nutrient level (0.25,

0.5 and 2%), and biochar level (0 or 2.5%) with each predictor as a factor, while also allowing for a possible effect for tree preheight in the model. All treatments had 1% lime and none contained LSM. Nutrients were at 0.25, 0.5 and 2.0% of the tailings weight and either had no biochar included or 2.5% biochar by weight. The most reduced model considered was an additive ANOVA model with nutrient source, nutrient level, and biochar level.

The third ANCOVA investigated the effects of nutrient and LSM on the response parameter. The initial MLR model considered included a two-way interaction using 6 treatments between the predictors of biosolids level (0.25, 0.5 and 2%) and LSM level (LSM or no LSM) (each predictor treated as a factor), while also allowing for a possible effect for tree preheight in the model. All treatments had 1% lime and 2.5% biochar. The 0.5% lime, fertilizer and no biochar treatments were not used. The most reduced model considered was an additive ANOVA model with nutrient level and LSM level.

Terms of the model were tested using Type III sums of squares F-tests (which are more powerful when covariates are present or groups are unbalanced) and statistically insignificant terms ($\alpha = 0.05$) were removed one step at a time. The final model was used to obtain contrasts using LS means and a Šidák correction to counteract the potential problem with multiple comparisons. Least squares means, or estimated marginal means, were computed for specific factors or combinations of factors to provide a fairer comparison if the data were unbalanced or covariates were present (Lenth, 2016).

A logistic regression with a generalized linear model (GLM) was performed for each question of interest to analyze the presence of treatment effects for the binary, response parameter: dead trees. Chi-squared tests were used instead of F-tests to reduce the full model considered and then

contrasts were estimated using LS means and applying a Šidák correction to counteract the potential problem with multiple comparisons.

Modifications were made to the basic analysis due to tree condition and when there were no values due to missing or omission of questionable data. Only trees that were living at the end of the experiment were included in the analysis for new needle dry weight and the needle elemental analyses. The leachate, pore water and salt extract analyses were modified for "zero" data values. In instances with high volumes of zero-valued responses due to the death of the plants, analyses were performed using only positive responses and the results pertain to living plants only.

The comparison of 1% lime and amendments was performed using SAS v. 9.4 (SAS Institute 2013). The other analyses are performed using R statistical software, version 3.5.0 (R Core Team, 2018) or higher. For exploratory data analysis, the ggplot2 R package (Wickham, 2009) was used to create all figures in this report with support from the cowplot R package (Wilke, 2017) to save plots. Additional statistical tools used in the analysis were found in the lsmeans R package (Lenth, 2016), emmeans R package (Lenth, 2020), and car R package (Fox, and Weisberg, 2019).

REFERENCES

Fox, J. & Weisberg, S. (2019). An R companion to applied regression, Third Edition. Thousand Oaks, CA: Sage Publications.

Lenth, R.V. (2016). Least-Squares Means: The R Package lsmeans. Journal of Statistical Software, 69, 1-33.

Lenth, R. (2020). emmeans: Estimated Marginal Means, aka Least-Squares Means. R packageversion 1.4.6. https://CRAN.R-project.org/package=emmeans

R Core Team. (2018). R: A Language and Environment for Statistical Computing. Vienna, Austria: R Foundation for Statistical Computing. https://www.R-project.org/.

Wickham, H. (2009). ggplot2: Elegant Graphics for Data Analysis. Springer-Verlag New York. http://ggplot2.org.

Wilke, C.O. (2017). Cowplot: Streamlined Plot Theme and Plot Annotations for 'ggplot2'. R package version 0.9.2. https://CRAN.R-project.org/package=cowplot.

SUPPLEMENTAL TEXT S3

Preliminary Amended Tailings Water Holding Capacity Study

In conjunction with the initial greenhouse experiment 1, a preliminary study was carried out to gravimetrically determine the water holding capacity for the Formosa tailings with different amendment treatments over time. Sample containers (three replicates per treatment) were prepared using the same tailings mixture batches prepared for greenhouse experiment 1 (GH1), with the same volume (500 ml) of mixtures per container. There were seven treatments: (1) tailings alone, (2) tailings + 1% lime, (3) tailings + 2% biosolids, (4) tailings + 1% lime + 2% biosolids, (5) tailings + 1% lime + 2% biosolids + 1% biochar, (6) tailings + 1% lime + 2% biosolids + 2.5% biochar, and (7) tailings + 1 % lime + 2% biosolids + 5% biochar.

Containers (the same Deepots as used in the greenhouse experiments) were lined with geotextile fabric (weed cloth) and filter paper to contain the treatment mixtures during the saturation process. Each Deepot, including the weed cloth and filter paper, was weighed to obtain the tare weight. In addition, the mean weight of water contained in wetted weed cloth and filter paper was obtained by weighing seven replicates of each both dry and following wetting. Also, three replicate subsamples of each of the GH1 treatment batches were weighed and dried for five days in a drying oven at 105°C to obtain the ambient (air dried) water content of the mixtures, determined as [(ambient weight - oven dry weight)/ambient weight]. The oven dry weight of the mixture in each of the Deepots was calculated by subtracting the tare weight of the pot, weed cloth, and filter paper from the total ambient weight to get the mixture ambient weight, and then multiplying by 1 minus the ambient water content.

Filled Deepots were placed in a holding rack and submerged in a tank of water for seven days to saturate the mixtures. Following saturation, each Deepot was removed from the water tank and allowed to drain for 1 minute and weighed to obtain the saturated (Day 0) weight. Deepots were also weighed after 24 hours to measure field capacity. The wet weight of the mixture in each Deepot was calculated as the total wet weight minus the tare weight of the pot, weed cloth, filter paper, and water content of the wetted weed cloth and filter paper. The gravimetric water content on a particular day was determined as [(wet weight - oven dry weight)/wet weight]. Deepots were kept at room temperature and allowed to drain, and reweighed after 2 and 12 days.

This study showed that adding biochar to the tailings, lime, and biosolids mixture clearly increased the water holding capacity (Figure 1). It also showed that the increase in water holding capacity was directly related to the % biochar addition. At field capacity (Time 0 or day 0 as in line 423) the tailings mixture with 5% biochar had nearly 30% water content, the 2.5% biochar had 25%, and the 1% biochar treatment had 22%. The four treatments without any biochar had the lowest water content (average just over 20%). The mixture with 5% biochar held about as much water after 12 days as the mixtures without biochar held after only one day.

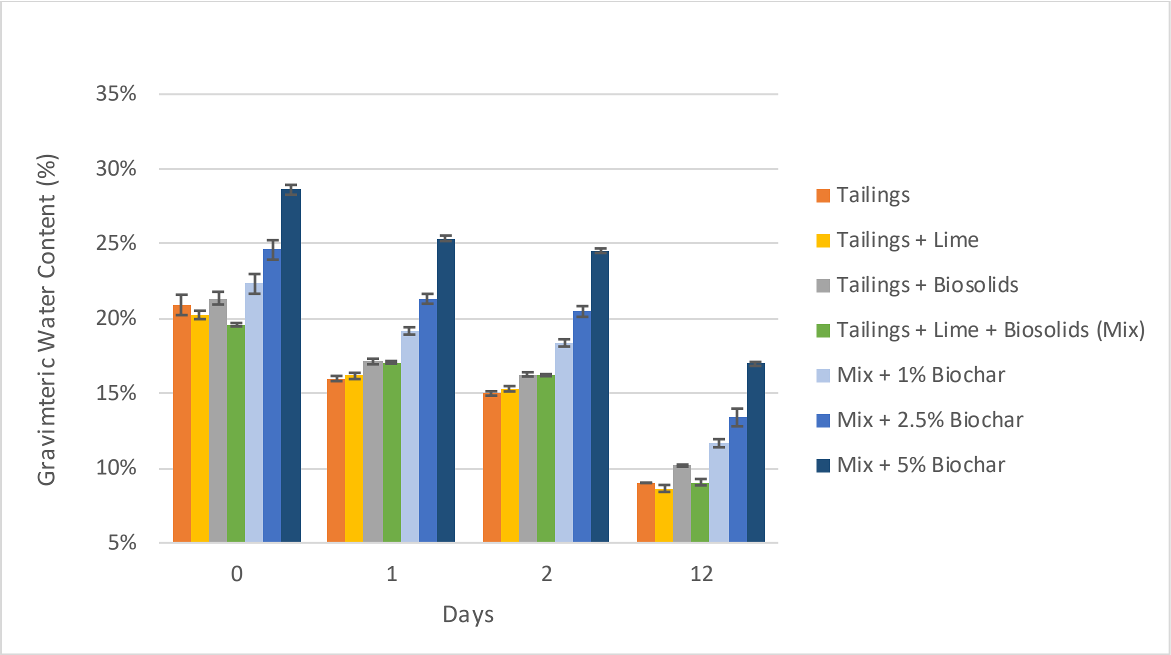

FIGURE 1 Gravimetric water content mean and standard error (n=3) for amended Formosa Mine tailings.
